# Structural Analysis of the Michael-Michael Ring Closure (MIMIRC) Reaction Products

**DOI:** 10.3390/molecules27092810

**Published:** 2022-04-28

**Authors:** Mabel M. Montenegro-Sustaita, Hugo A. Jiménez-Vázquez, Elena Vargas-Díaz, J. Enrique Herbert-Pucheta, L. Gerardo Zepeda-Vallejo

**Affiliations:** Departamento de Química Orgánica, Instituto Politécnico Nacional, Escuela Nacional de Ciencias Biológicas, Prolongación de Carpio y Plan de Ayala s/n, Col. Santo Tomas., Alc. Miguel Hidalgo, Ciudad de México 11340, Mexico; mabel.montenegro2019@gmail.com (M.M.M.-S.); hjimenezv@ipn.mx (H.A.J.-V.); mvargasd@ipn.mx (E.V.-D.); jherbertp@ipn.mx (J.E.H.-P.)

**Keywords:** decalin/hydrindane derivatives, double Michael addition, methyl acrylate, cycloalkanone enolates, stereochemical outcome

## Abstract

A representative number of decalin and hydrindane derivatives **2a**–**l** were prepared in 11–91% yield by means of a cascade reaction of cyclohexanone/cyclopentanone enolates and methyl acrylate through a Michael–Michael ring closure (MIMIRC) process. The relative stereochemistry of the four stereogenic centers formed in all products was determined by analyzing the vicinal coupling constants from the ^1^H NMR and X-ray crystallography. Such a stereochemical outcome was corroborated by conformational analysis supported by DFT calculations and simulating the ^1^H NMR spectra of representative products. All products showed the same relative stereochemistry at C-1 and C-8a, while at C-3 and bridgehead carbon C-4a, configurational changes were observed. The present results provide some insights about the scope and limitations of the triple cascade reaction between cycloalkanone enolates with methyl acrylate. This synthetic protocol is still a simple and very practical alternative to generate decalin and hydrindane derivatives with great structural diversity.

## 1. Introduction

**Figure 1:**
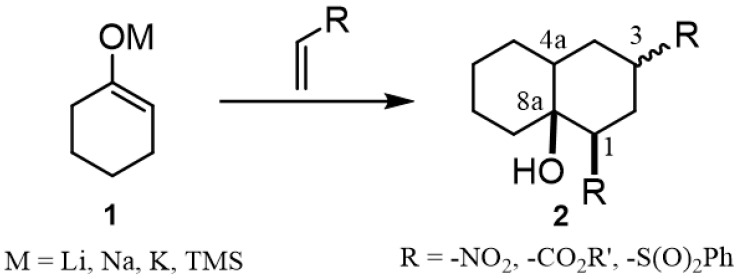
General approach for the synthesis of decalin derivatives via Michael’s double addition of cycloalkanone enolates over conjugated vinylic systems.

Decalins and hydrindane frameworks are structural motifs present in a large number of natural polycyclic compounds produced by a wide diversity of plants and microorganisms [1,2]. The synthesis of decalin derivatives has been a research topic that dates back several years due to their diverse and significant biological properties, such as their antifungal [3], antibacterial [4], anticancer [5,6] and immunosuppressive [7] activities. Among the wide variety of synthetic strategies developed to prepare them [8], Robinson annulation [8], inter-/intramolecular Diels–Alder cycloadditions [9] and Michael or Aldol reactions [10,11] continue to be widely used due to their simplicity and effectiveness. In fact, the latter synthetic approach could be considered as a practical synthetic alternative due to the availability of a wide variety of cyclic and acyclic enolates and conjugated vinylic systems capable of providing a rich structural variety of decalin derivatives. Figure 1 shows the representative approach for the synthesis of decalin derivatives through the addition of enolates over conjugated vinylic systems used as starting material. 

In particular, it appears that the preparation of decalin derivatives by Michael–Michael ring closure (MIMIRC) double addition [10,11] has partially been overlooked due to the large number of novel synthetic alternatives that were developed for the same purpose in the last 20 years [12,13,14,15,16]. However, since the pioneering work of Posner and colleagues [10,11], the synthetic value of this protocol due to its high convergence, atom economy and simplicity of the reaction conditions has been demonstrated, thus remaining as a competitive alternative to produce decalins with a rich assortment of functional groups.

While exploring this protocol in our research group by reacting cycloalkanone lithium enolates with methyl acrylate, it was observed that the stereochemistry of products at C-3 and C-4a demonstrates a lack of consistency, giving rise to a mixture of up to four possible diastereomeric products. Contrary to this, the relative stereochemistry at C-1 and C-8a is uniformly maintained as *cis*. This behavior was also systematically observed in the literature [17,18,19,20]. It should be noted that C-4a defines the *cis* or *trans* fusion of the newly formed cycle; however, discerning between these geometries is often not so trivial in some cases. Accordingly, we sought to prepare a representative series of decalin and hydrindane derivatives to analyze the relative stereochemistry at C-3 and C-4a of the main isolated products. The present structural analysis was mainly performed by suggesting transition states explaining the substituent effects on the stereochemistry of the products, as well as simulating the ^1^H NMR spectra of some representative compounds. These results could be useful to support future MIMIRC synthetic protocols.

## 2. Results and Discussion

### 2.1. Preparation of Decalin and Hydrindane Derivatives

A set of cyclohexanones with structural diversity was selected for the first ten assays (Table 1). Entry 1 shows the reaction of lithium enolate of cyclohexanone with methyl acrylate in anhydrous THF at −78 °C under nitrogen atmosphere. After column chromatography of the crude reaction, the presence of a decalin derivative was revealed, which in turn was fully characterized as *trans*-decalin **2a** (vide infra). Under similar reaction conditions, the respective lithium enolates of substituted (**1b**–**d**, **1f**, **1j**; Table 1, entries 2–4, 6 and 10) and conjugated (**1e**, **1h** and **1i**; Table 1, entries 5, 8 and 9) cyclohexanones were also condensed with methyl acrylate, affording the corresponding decalin derivatives in a range of 11–77% yield after column chromatography. 

Some structural features of the obtained products can be mentioned. It can be observed that decalins **2a** and **2b** have the same stereochemistry at C-1, C-3, C-4a and C-8a. In contrast, a particular structural feature of decalins **2c**–**e** and **2g**–**i** is the β stereochemistry at C-3, which allowed the formation of a lactone ring between the methylester at C-3 and the hydroxyl group attached to C-8a. It is worth noting that despite a 3:1 diastereomeric mixture of *trans*- and *cis*-2,6-dimethylcyclohexanone **1d**, respectively, being used (Table 1, entry 3), decalin **2d** bearing both methyl groups in a *cis* relationship was isolated as a major product. It can also be observed that a C-2 epimeric mixture of menthone **1g** was used, thus affording a complex mixture of at least three lactonized decalins **2g** (Table 1, entry 7), as is shown in its respective ^1^H NMR spectrum (See Supplementary Materials). Besides the inherent lack of a OMe signal in the ^1^H NMR spectrum of lactones, differentiation between non-lactonized and lactonized decalins can also be performed according to the ^13^C NMR chemical shifts (δ) of C-8a: δ~67–75 for non-lactonized (**2a**, **2b**, **2e**, **2f**, **2j**) and δ~80–85 for lactonized (**2c**, **2e** and **2g**–**i**) decalins. This fact can be useful to differentiate a lactone from a carboxylic acid derivative, as occurred in decalin **2e**, which likely was lactonized and later opened during its purification through silica gel column chromatography. In this sense, C8a in this decalin appears at δ 66.9, clearly belonging to a non-lactonized decalin.

**Figure 2:**
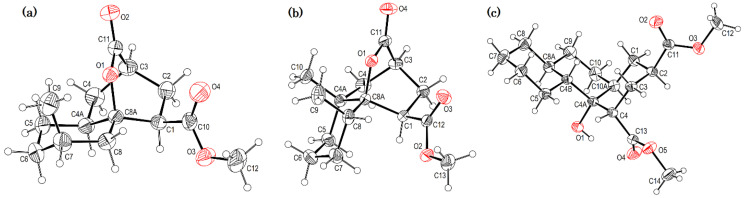
ORTEP projections of (from left to right): (**a**) *trans*-decalin derivative **2c**; (**b**) *cis*-decalin derivative **2d**, and (**c**) perhydrophenanthrene derivative **2j**. Displacement ellipsoids for the non-hydrogen atoms are drawn at 30% probability.

Crystals suitable for X-ray diffraction analysis were obtained for lactonized decalins **2c** and **2d** and for perhydrophenantrene derivative **2j** (Figure 2). Lactones revealed two interesting structural features: (a) all substituents have a β relationship around the bicyclic system, and (b) they can differ in the stereochemistry of the bridgehead carbon at C-4a position; while **2c** is a *trans*-decalin, **2d** is a *cis*-decalin.

The above examples represent clear evidence of the great structural diversity that can be obtained through this synthetic protocol just by using different cycloalkanones. In addition, esters (or lactone), ketal (**2f**) and double bonds (**2e**, **2h** and **2i**) present in products should allow us to further functionalize them to increase their structural assortment. It is noteworthy that chiral naturally occurring ketones as well as polycyclic ketones should lead to optically active products or polycyclic structures in just one-pot procedure, respectively, which could be harder to achieve by other synthetic methods. Such is the case of pulegone **1h** and carvone **1i,** which led to chiral decalins **2h** and **2i**, respectively, and bicyclic ketone **1j** afforded the perhydrophenantrene derivative **2j**. The structural diversification of acrylates should provide a greater richness of functional groups around the bicyclic structures.

On the other hand, the reaction of cyclopentanone enolates **1k** and **1l** (Table 1, entries 11 and 12) with methyl acrylate afforded *cis*-hydrindanes **2k** (75%) and **2l** (91%), respectively. As can be observed, a similar stereochemical outcome was obtained as compared with some decalins: a *cis* relationship at C-1 and C-7a (C8a for decalins), as well as α (**2l**) or β (**2k**) stereochemistry at C-3.

### 2.2. A Theoretical Structural Analysis

**Figure 3:**
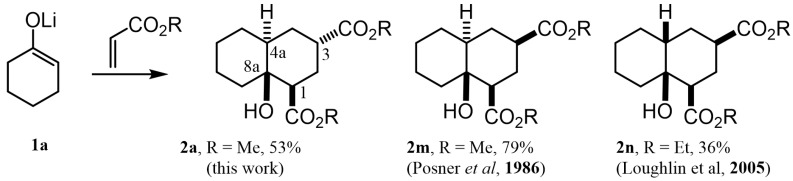
Decalin diastereomers obtained by MIMIRC reaction between cycloalkanone lithium enolate and methyl/ethyl acrylate described for different research groups [17,20].

As can be observed, decalin **2a** possesses four stereogenic centers. This means that this reaction should give rise to up to eight NMR-distinguishable diastereoisomers (Figure 2); however, **2a** was obtained as the major one (43%). After analyzing its ^1^H NMR spectrum (see Supplementary Materials), it was found that its relative stereochemistry at C-1, C-3, C-4a and C-8a is β, α, α and β, respectively, as was obtained by the scalar couplings of H-1/H-2ax, H-2eq (J_H-1,H-2ax_ = 13.0, J_H-1/H-2eq_ = 4.0 Hz), H-2ax,H-2eq/ H-3 (J_H-3,H-2ax_ = 5.0, J_H-3/H-2eq_ = 2.1 Hz) and H-3/H-4ax,H-4eq (J_H-3,H-4ax_ = 5.0, J_H-3/H-4eq_ = 2.0 Hz). In contrast with this finding, different diastereomers have been described [17,20] as the major product for the same MIMIRC reaction under similar conditions (Figure 3). It can be observed that the stereochemical outcome is consistent at C-1 and C-8a, while configurations at C-3 and C-4a change in a seemingly random event. Therefore, these stereochemical changes are responsible for producing a mixture of two to four possible diastereoisomers, and this could be one of the reasons why the MIMIRC reaction has a range of low to medium overall chemical yields for the major product.

**Figure 4:**
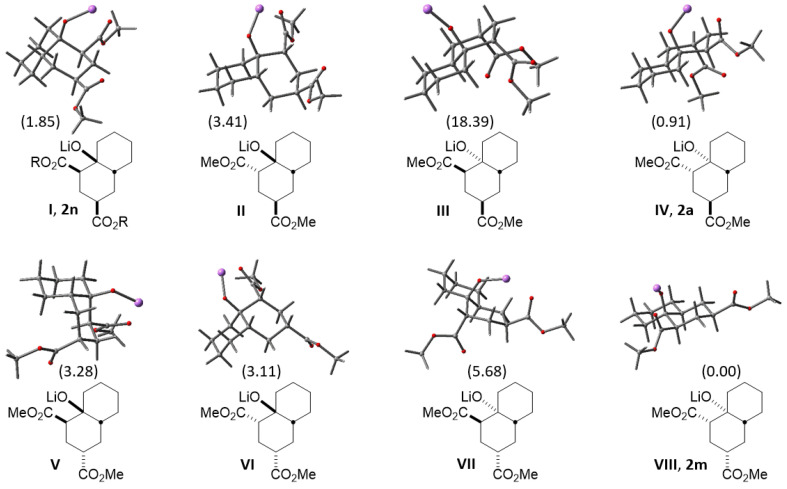
Plain and optimized 3D structures of the eight possible diastereomers (**I**–**VIII**) obtained by MIMIRC reaction between cyclohexanone lithium enolate and methyl acrylate. These projections show the most stable conformer for each diastereomer and its relative energies in brackets. Diastereomers **2n** (R = Et), **2a** and **2m** correspond to the protonated version of compounds **I**, **IV** and **VIII**, respectively. The energy shown for **2n** is for the dimethyl ester (R = Me).

According to the above, it was considered useful to know the relative stability of the eight possible decalin diastereoisomers obtained by reacting cyclohexanone enolate and methyl acrylate (Figure 4). Thus, each diastereomer was subjected to a conformational search using PC Spartan Pro [21] and Molecular Mechanic Force Field [22]. The resulting conformers were optimized by using Gaussian 09 program [23] at the HF-631G* level of theory. Finally, each minimum was submitted to geometry optimization using functional ωB97X-D [24] along with the 6-31 + G(d,p) basis set. All calculations were performed on the corresponding lithium alkoxides. Table 2 shows the obtained relative energies (ΔG, kcal/mol) for the four to five most stable conformers for diastereoisomers **I**–**VIII** (found for each diastereomer below 20 kcal/mol), and in Figure 4 the plain structures and geometry of the most stable conformers of the eight possible diastereomers can be observed, along with the relative energy shown in brackets. It should be noted that diastereomer **VIII** is the most stable, followed by **IV** and **I**, whose structural frameworks belong to compounds **2m**, **2a** and **2n**, respectively, their protonated version.

Assuming that thermodynamic equilibrium is reached in the formation of the eight diastereomeric alkoxides, it would be possible to estimate the ratio in which these compounds would be obtained from their relative stabilities. Table 3 shows the relative population (%) for the two most stable conformers of diastereoisomers **2a** (20%), **2n** (~3%) and **2m** (~77%) obtained from the Boltzmann distribution law at 25 °C. As can be seen, the expected population of decalin **2m** (~77%) is close to the experimental yield (79%) previously described [15]; however, other experimental results reveal the presence of decalins **2a** (43%, this work) and **2n** (36%) [18]. This behavior may be due to subtle variations in reaction conditions carried out by each research group, which likely influence some step of the cascade reaction. Therefore, this theoretical information could be useful to homogenize criteria about the reaction conditions required to achieve optimized and coincident results. 

**Figure 5:**
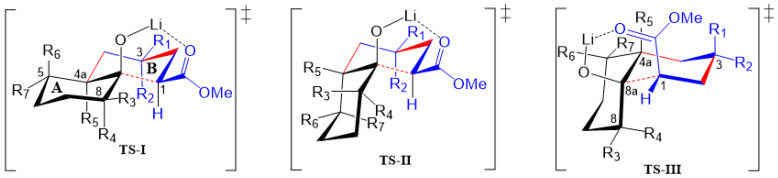
Transition states (TSs) proposed for the MIMIRC reaction between methyl acrylate and lithium enolate of substituted cycloalkanones. Substituent patterns will determine the stereochemistry of the obtained products.

On the other hand, it is feasible to argue that during the first Michael addition, diastereomeric transition states (TSs) with nearby energies can be formed when using cyclohexanone lithium enolate, affording two possible diastereoisomers due to the formation of the two first stereogenic centers. However, the following stage (consecutive 1,4- and 1,2-addition) of the triple cascade reaction involves a more rigid Zimmerman–Traxler-type TS [25], which could adopt any of the three *chair*–*chair* configurational/conformational arrangements shown in Figure 5. These three chelated TSs explain the *cis* relationship between OH and CO_2_Me at C-1 as was mentioned above. As can be seen, in the last step the *cis*/*trans* stereochemistry will be determined, in which substituents at ring A (if present) and at the incipient ring B could influence the stereochemistry of the products. Firstly, it should be emphasized that the following reasoning applies for non-lactonized decalins and for those prior to lactonization, where applicable.

Thus, **TS-I** will preferably conduct *trans*-decalin **2m** when R_1_ = CO_2_Me(Et), R_2_ = R_3_ = R_4_ = R_5_ = H, and will afford *trans*-decalin **2a** (R_2_ = CO_2_Me(Et), R_1_ = R_3_ = R_4_ = R_5_ = H) in lower yield than **2m** because in **2a,** R_2_ shows 1,3-diaxial interaction with H-1 and R_5_ (H-4a, or alkyl group). In turn, **TS-II** gives *cis*-decalin **2n** (R_1_ = CO_2_Me; R_2_ = R_3_ = R_4_ = R_5_ = H), which is obtained in the lowest yield. This reasoning makes sense, and it is supported by the relative energy of diastereomers **VIII**, **IV** and **I** determined by theoretical calculations (Figure 3), which should be formed through **TS**-**I** (R_1_ = CO_2_Me), **TS**-**I** (R_2_ = CO_2_Me) and **TS**-**II** (R_1_ = CO_2_Me), respectively. In other words, it can be assumed that in the absence of substituents on the cycloalkanone ring (R_3–7_ = H), *trans* decalins will be favored over *cis* decalins.

The proposed TSs help to visualize the steric effects of substituents at ring A that can drive the stereochemistry of the obtained products. Firstly, it can be assumed that cycloalkanone bearing an α-substituent (**1b**, **1d**, **1g**, **1j** and **1l**) will produce the kinetic lithium enolate with LDA, which will react with methyl acrylate, affording decalins/hydrindanes substituted at C-8/C-7, respectively. In all TSs (Figure 5), such substituents may have β (R_3_) or α (R_4_) orientation when C-8 has an *sp^3^* hybridization; however, the results shown in Table 1 reveal that β orientation is preferred over α orientation, giving **2b**, **2d**, **2g** (at least one diastereomer of the three formed), **2j** and **2l**. Accordingly, **TS**-**I** reveals that any alkyl substituent will prefer β-equatorial orientation (R_3_) over α-axial orientation (R_4_), as the later orientation shows 1,3-axial interactions with H-1, H-4a (R_5_) and H-6α. It can also be observed in TS-I that the larger the size of R_3_, the more important the 1,3-diquatorial interaction with the CO_2_Me group at C-1 is. Moreover, if this condition is met, the formation of a *cis*-decalin via **TS**-**II** or **TS**-**III** may be preferred, as described by Jaafar et al. [19]. Although this 1,3-diequatorial interaction may also be present between R_4_ and the CO_2_Me group at C-1 in **TS**-**II**, this TS would be less available than **TS-I** due to the expected higher stability of a *trans*-decalin-type TS compared with a *cis*-decalin-type TS. 

Besides substituents at C-8/C-7 (decalin/hydrindane), substituents at C-4a and C-5 would also influence stereochemistry of products by inducing the formation of any of the TSs proposed. For instance, the formation of *cis*-decalin precursor of lactone **2d** can be explained through **TS-II**, since it has the lowest number of 1,3-diaxial interactions (R_3_ = R_5_ = Me, R_1_ = CO_2_Me; R_2_ = R_4_ = R_6_ = R_7_ = H) as compared with **TS-I** in either of the diastereomeric combinations R_3_ = R_5_ = Me, or R_4_ = R_5_ = Me. In turn, an α substituent (R_7_ = alkyl) at C-5 is expected to induce the formation of a *trans*-decalin via **TS-I**, whereas if it is at β (R_6_ = alkyl) it will favor a *cis*-decalin through **ET**-**II**. The latter case explains formation of decalins **2g** and **2h**, and the former afford decalin **2i**. On the other hand, the absence of substituents at ring A or the presence of substituents at C-6 or C-7 normally will afford *trans*-decalin derivatives, as compounds **2a**, as well as **2c** and **2f**, respectively. In general, these three TS models reasonably fit the results shown in Table 1.

### 2.3. A ^1^H NMR-Based Structural Analysis of Decalin and Hydrindane Derivatives

**Figure 6:**
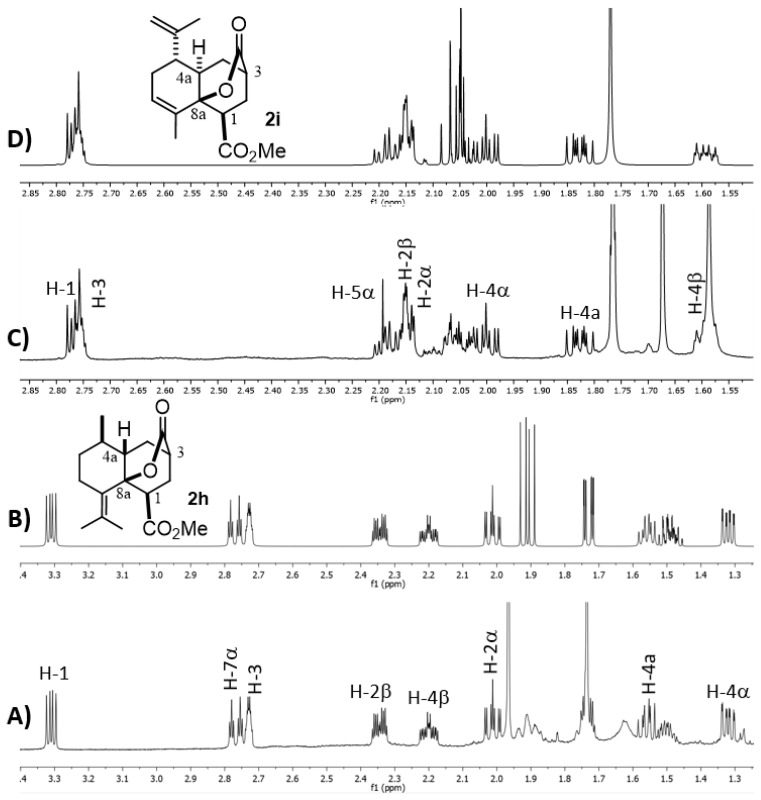
Experimental (**A**,**C**) and simulated (**B**,**D**) ^1^H NMR spectra of **2h** and **2i**, respectively. Full ^1^H NMR simulated parameters were considered only for shown protons.

**Figure 7:**
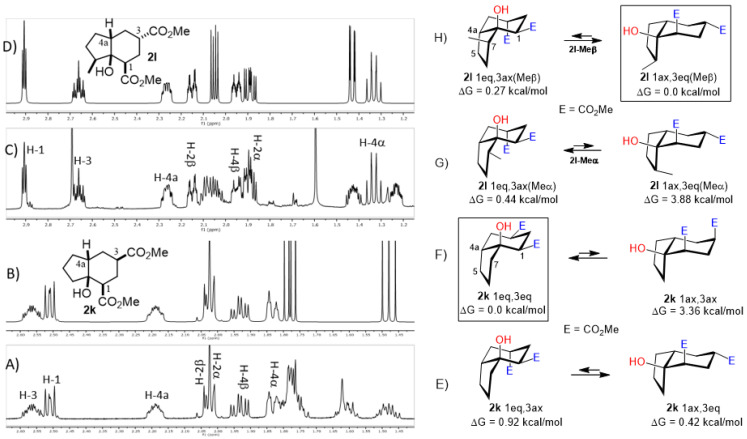
Experimental (**A**,**C**) and simulated (**B**,**D**) ^1^H NMR spectra of hydrindanes *cis*-**2k** and *cis*-**2l**, respectively. Full ^1^H NMR simulated parameters were considered only for the shown protons. (**E**–**H**) show the relative energies of conformers for *cis*-hydrindanes **2k** and **2l**, respectively, which illustrate the conformational influence of CO_2_Me and Me groups attached at C-3 and C-7, respectively. Structures inside rectangles belong to the most stable conformers.

Finally, to complete the present structural study, we decided to further analyze the ^1^H NMR parameters of the MIMIRC products since they are often decisive in establishing the relative stereochemistry of derivatives that did not provide suitable crystals for X-ray diffraction. It is well known that this goal can be achieved by correlating dihedral angles (*ϕ*) with scalar coupling constants (*J*) between vicinal protons [26,27]. However, when strong coupling or signal overlapping occurs, it is a challenge to know the precise values of δ and *J*, thus complicating the configurational/conformational analysis. Under this circumstance, simulating the ^1^H NMR spectra represents a reliable alternative to knowing the NMR parameters that helps to reveal the relative stereochemistry of a given molecule [28,29,30], as well as to correctly assign its corresponding spectra. Accordingly, in the search to establish the relative stereochemistry at C-1, C-3, C-4a and C-8a for each product, it was useful to simulate the ^1^H NMR spectrum comprising signals of protons of the newly formed six-membered ring (H-1, H-2αβ, H-3, H-4αβ and H-4a) of a representative number of derivatives (Figure 6 and Figure 7 and Supplementary Materials). Thus, Figure 6 shows the experimental and simulated ^1^H NMR spectra of lactones *cis*-**2h** and *trans*-**2i**, which show a particular chemical shift and multiplicity pattern related to the configurational relationship of substituents and *cis* or *trans* ring fusion. Firstly, the multiplicity of the downfield isolated signals of H-1 and H-3 provides explicit structural information about their relative configuration. As expected, in all lactonized decalins, these protons show a consistent pattern of multiplicity due to the restricted conformational freedom on this part of the molecule.

On the other hand, the relative *cis*/*trans* configuration at C4a-C8a should be carefully analyzed through scalar coupling constants (*J*) in fragment CH_2_-4/H-4a/CH(R)-5. For instance, *trans* decalone **2i** shows the following dihedral angles (*ϕ*) and expected *Js*: H4α-H4a (20°, *J* = 9.3 Hz), H4β-H4a (138°, J = 7.6 Hz) and H4a-H5α (171°, *J* = 11.5 Hz), whereas the same NMR parameters for *cis* decalone **2h** are H4α-H4a (238°, *J* = 6.6 Hz), H4β-H4a (345°, *J* = 9.8 Hz) and H4a-H5α (175°, *J* = 12.1 Hz). A similar analysis for decalins **2a**, **2e** and **2d** was performed, whose simulated spectra and full expected and simulated NMR parameters are shown in the Supplementary Materials section. It should be mentioned that the *cis-* or *trans*-stereochemistry around the C8a-C4a bond of decalins **2h**, **2i** and **2j** was fully supported by nOe difference experiments by selective irradiation of H-1 and/or H-3 on **2h** and **2i**, as well as H-2 and/or H-4 on **2J** (Figures S23 and S27).

A special emphasis was placed on hydrindanes **2k** and **2l,** since their simulated NMR parameters led to different conformations even though they possess the same *cis*-stereochemistry at C4a-C7a. In order to support this finding, we sought to determine the relative stability of *cis*-hydrindane **2k,** considering two possible epimers at C-3, giving a couple of conformers for each epimer. Figure 7E,F show the structure for each couple of conformers as well as their relative energy (DFT/PBEPBE/DGDZVP//HF/631G*) [31,32], where it can be observed that conformer **2k**-1eq,3eq possesses the lower energy. The resulting ^1^H NMR simulated spectrum (Figure 7B) is consistent with dihedral angles expected for the above molecular geometry. It should be noted that if there is no substituent on the 5-membered ring, the preferred product is **2k**-1eq,3eq, since the other structures contain at least one CO_2_Me group in the axial position. In turn, hydrindane **2l** has a Me group at C-7, and so the resulting pair of conformers for each epimer were considered in this case. In Figure 7G,H are shown the mentioned coupled of conformers, from which it can be observed that conformer **2l**-1ax,3eq is the most stable. Such geometry is also consistent with the ^1^H NMR simulated spectrum shown in Figure 7D. From these results, it is possible to argue the following: if the CO_2_Me group at C-3 is α, the 1,3-diaxial interaction between it and C-5 is destabilizing (**2k**-1ax,3eq vs. **2k**-1eq,3ax); however, if there is an α alkyl group at C-7 and an α CO_2_Me group at C-3, the dominant conformer is the one where the CO_2_Me group is axial (**2l**-1eq,3ax(Meα) vs. **2l**-1ax,3eq(Meβ)). On the other hand, if the alkyl group at C-7 is β, the CO_2_Me group adopts an equatorial position (**2l**-1eq,3ax(Meβ) vs. **2l**-1ax,3eq(Meβ)). These results are consistent with the transition states proposed in Figure 5. Furthermore, a nOe difference experiment provided information on the β stereochemistry of methyl group at C-7, as an enhancement in H-7 was observed after the selective irradiation of H-1. 

## 3. Conclusions

The present results provide useful information about the structural features of decalin and hydrindane derivatives obtained from the Michael–Michael addition of cycloalkanone enolates on methyl acrylate. This procedure offers a short alternative to reach a variety of such derivatives with a rich structural diversity due to the wide availability of cycloalkanone and acrylate derivatives. Although the reaction generates polymerization by-products and epimeric mixing at C-3 or C-4a, on average, 30–40% of the major products are obtained among eight possible diastereoisomers, which is equivalent to about 70% yield for each of the three consecutive reactions occurring in the whole process. As expected, kinetic enolates were formed when α-monosubstituted cyclohexanones were used (**1b** and **1g**; Table 1, entries 2 and 7), as was corroborated through their corresponding isolated products (**2b** and **2g**, respectively). Further, a relative *cis* stereochemistry at C-1 and C-8a was always obtained, while epimeric possibilities were observed at C-3 and C-4a. For instance, when β-stereochemistry of the CO_2_Me group at C-3 is obtained, this allowed lactonization with the OH group at C-8a, yielding lactones **2c**, **2d**, **2g–i**, whilst epimers at C-4a give raise to *trans*- or *cis*-decalin/hydrindane derivatives. Simulating the ^1^H NMR spectra of products, considering the protons where the new stereogenic centers are generated, helps to determine the relative stereochemistry of those derivatives that do not provide suitable crystals for X-ray diffraction. 

## 4. Materials and Methods

### 4.1. General Information

^1^H and ^13^C NMR experiments were performed at 500 and 125 MHz (Varian NMR System), or at 600 and 150 MHz (Bruker Avance III), respectively, using CDCl_3_ as the solvent and TMS as the internal reference. ^1^H NMR spectra simulations were performed by using MestReNova^TM^ software v. 12.0.0 (Mestrelab Research S.L.), and they correlation between vicinal dihedral angles and *^3^J*_1H-1H_ was calculated by using MestReJ [30] and Altona programs [29]. High resolution mass spectra (HRMS) were obtained in a Jeol JSMGCMate II mass spectrometer, using electron impact techniques (70 eV). X-ray data were collected on an Oxford Diffraction Xcalibur S single-crystal X-ray diffractometer. Optical rotations for compounds **2h** and **2i** were acquired with a JASCO P-2000 polarimeter in CH_2_Cl_2_ solutions. TLC analyses were performed on silica gel plates, visualized with UV (254 nm) or were developed with a spray of phosphomolybdic acid solution. Lithium diisopropylamide (2.0 M in hexane) was used as purchased (Sigma-Aldrich, Burlington, MA, USA). All reactions were carried out under nitrogen in anhydrous solvent. All glassware was dried in an oven prior to use and all commercially available compounds were used without further purification. Melting points are not corrected. Tetrahydrofuran was distilled from benzophenone ketyl-sodium indicator under N_2_ atmosphere prior to use. n-Hexane and ethyl acetate for chromatographic separations were distilled before use. 

### 4.2. Theoretical Calculations

Geometries of the lithium alkoxide decalins **I**–**VIII** were generated with PC Spartan Pro 1.0.5 [21]. For the *cis* decalins, the two possible decalin conformations were considered as independent systems. For each system, a conformational search was carried out using the conformational search method included in PC Spartan Pro, using the Merck Molecular Force Field (MMFF) [22]. Duplicates were eliminated and the unique geometries were optimized with Gaussian 09 [23] at the HF/6-31G* level of theory. The surviving minima were optimized using the ωB97X-D functional [24] combined with the 6-31 + G(d,p) basis set. All optimizations with Gaussian 09 were carried out using the OPT = TIGHT option. In addition, DFT calculations were carried out using the INT(GRID = ULTRAFINE) option. In all cases, vibrational frequencies were calculated at the optimized geometries at the same level of theory to verify that they corresponded to minima (no imaginary frequencies) and to obtain free-energy corrections to the electronic energies at 298 K. A similar protocol was used to perform the conformational analysis of hydrindanes **2k** and **2l**. Thus, after the conformational search performed with PC Spartan Pro (MMFF), conformers under 10 kcal/mol were further optimized at the HF/631G* level of theory with Gaussian 09. Afterwards, each conformer was optimized by using DFT level of theory and functional/basis set PBEPBE [26]/DGDZVP [32]. 

### 4.3. General Procedure for the Preparation of Decalin Derivatives ***2a–j*** and Hydrindane ***2k,l*** by Reacting Cycloalkanone Enolates (***1a–k***) with Methyl Acrylate

To a cooled solution (−78 °C) of the corresponding ketone (**1a**–**k**) (1.0 mol equiv) in THF (5 mL) was added LDA solution 2.0 M in n-hexane (1.1 mol equiv) dropwise. The mixture was stirred at −78 °C for 30 min; then, a solution of methyl acrylate in THF (1.0 mol equiv) was slowly added. The reaction mixture was kept at −78 °C for 1 h and then warmed up to room temperature. The mixture was quenched after 3–12 h with a saturated aqueous solution of NH_4_Cl (10.0 mL) and then was extracted with ethyl acetate (3 × 25 mL). The combined organic layers were washed with brine, dried over anhydrous Na_2_SO_4_, filtered, and concentrated under vacuum. The resulting residue was purified by column chromatography over silica gel using *n*-hexane/EtOAc (9:1) as an eluent, to afford the corresponding products (**2a–l**).

#### 4.3.1. Dimethyl 8a-Hydroxydecahydronaphthalene-1,3-Dicarboxylate (**2a**)

Following the general procedure, a solution of 500 mg (5.0 mmol) of cyclohexanone **1a** in anh. THF was treated with 1.43 mg (6.6 mmol) of LDA. To the resulting enolate was added methyl acrylate (10.59 mmol) to afford 318 mg (47.0%) of **2a** as a white solid. Mp = 32.5–34 °C. ^1^H NMR (600 MHz, CDCl_3_) δ 3.74 (s, 3H, OMe), 3.73 (s, 3H, OMe), 3.07 (s, 1H, OH), 2.79 (dddd, *J* = 5.3, 5.0, 2.5, 1.8 Hz, 1H, H-3), 2.62 (dd, *J* = 12.7, 4.1 Hz, 1H, H-1), 2.20–2.07 (m, 2H, H2ax, H2eq), 1.84 (td, *J* = 13.0, 5.5 Hz, 1H, H-4ax), 1.72 (m, 1H, H-4ec), 1.76–1.18 (m, 9H, H-4a, H-5ax, H-5eq, H-6ax, H-6eq, H-7ax, H-eq, H-8ax, H-8eq). ^13^C NMR (151 MHz, CDCl_3_) δ 176.8 (C-9), 175.4 (C-11), 68.7 (C-8a), 51.7 (OMe), 51.7 (OMe), 48.54, 40.19, 38.36, 37.24, 29.71, 28.72, 27.94, 26.23, 25.94, 21.37. 

#### 4.3.2. Dimethyl 8a-Hydroxy-5-Methyl-8-(Propan-2-Ylidene) Decahydronaphthalene-1,3-Dicarboxylate (**2b**)

Following the general procedure, a solution of 100 mg (0.89 mmol) of 2-methylcyclohexanone **1b** in anh. THF was treated with 0.19 mg (0.89 mmol) of LDA. To the resulting enolate was added methyl acrylate (1.78 mmol) to afford 0.105 g (42%) of a diastereomeric mixture of **2b** as a yellowish syrup. 

Major diastereomer **2b**. IR (KBr): 3507.2, 2033.4, 2861.1, 1737.7, 1711.8 cm^−1^. ^1^H NMR (600 MHz, CDCl_3_) δ 3.64 (s, 3H, OMe), 3.62 (S, 3H, OMe), 2.67 (m, 1H, H-3), 2.52 (dd, *J* = 12.8, 4.1 Hz, 1H, H-1), 2.01 (m, 1H, 2α), 1.94 (td, 12.8, 5.0 Hz, 1H, 2β), 1.79 (dt, *J* = 13.3, 5.4 Hz, H-4β), 1.74-1.10 (m, 8H, H-4a, H-5ax, H-5eq, H-6ax, H-6eq, H-7ax, H-eq, H-8), 0.63 (d, *J* = 6.7 Hz, 3H, H-13). ^13^C NMR (151 MHz, CDCl3) δ 174.80 (C-11), 172.81 (C-9), 72.39 (C-8a), 52.02 (OMe), 51.79 (OMe), 45.94, 45.69, 42.13, 41.33, 38.27, 37.42, 31.71, 29.15, 28.53, 28.36, 25.83, 24.73, 16.39 (C-13). HRMS (EI^+^) calcd for C_15_H_24_O_5_ 284.1616. Found (MH^+^) 284.1624.

#### 4.3.3. Methyl 7-Methyl-10-Oxooctahydro-2H-4a,2-(Epoxymethano)Naphthalene-4-CARBOXYLATE (**2c**)

Following the general procedure, a solution of 100 mg (0.89 mmol) of 3-methylcyclohexanone **1c** in anh. THF was treated with 0.19 mg (0.89 mmol) of LDA. To the resulting enolate was added methyl acrylate (1.78 mmol) to afford 176 mg (77%) of **2c** as a white solid. Mp = 122–123 °C. IR (KBr): 3735.7, 522.3, 2929.5, 1731.9, 1715.4, 1436.1 cm^−1^. ^1^H NMR (600 MHz, CDCl_3_) δ 3.74 (s, 3H, OMe), 3.13 (dd, *J* = 10.7, 5.7 Hz, 1H, H-1), 2.72 (m, 1H, H-3), 2.12–2.23 (m, 3H, H-2β, H-4β, H-7), 2.07 (ddd, *J* = 13.4, 10.6, 2.8 Hz, 1H, H-2α), 1.98 (ddd, *J* = 14.2, 2.81, 1.5 Hz, 1H, H-8β), 1.87 (dd, *J* = 14.2, 6.1 Hz, 1H, H-8α), 1.81 (m, 1H, H-4a), 1.72 (dbq, *J* = 13.8, 4.0 Hz, 1H, H-5α), 1.62 (m, 1H, H-6α), 1.50 (m, 1H, H-6β), 1.35 (qd, *J =* 13.1, 4.0, 1H, H-5β), 1.22 (ddd, *J* = 13.1, 8.6, 1.4 Hz, 1H, H-4α), 1.06 (d, *J =* 7.6 Hz).^13^C NMR (151 MHz, CDCl_3_) δ 175.3 (CO_2_Me), 172.9 (CO_2_), 82.5 (C-8a), 52.0 (OMe), 41.9 (C-1), 41.4 (C-4a), 37.9 (C-8), 35.3 (C-3), 30.9 (C-6), 30.0 (C-4), 29.9 (C-2), 26.6 (C7), 25.1 (C-5), 20.2 (CH_3_). HRMS(EI^+^) calcd for C_14_H_20_O_4_ 252.1362. Found (MH^+^) 252.1358.

#### 4.3.4. Methyl 5,8a-Dimethyl-10-Oxooctahydro-2H-4a,2-(Epoxymethano)Naphthalene-4-Carboxylate (**2d**)

Following the general procedure, a solution of 0.3 mL (2.3 mmol) of *trans*-2,6-dimethylcyclohexanone **1d** in anh. THF was treated with 0.5 mg (2.4 mmol) of LDA. To the resulting enolate was added methyl acrylate (4.7 mmol) to afford 350 mg (52%) of **2d** as a colorless solid. Mp 136–137 °C. IR (KBr): 3464.1, 2947.6, 1760.9, 1738.6 cm^−1^. ^1^H NMR (600 MHz, CDCl3) δ 3.70 (s, 3H, -OMe), 3.42 (dd, *J* = 10.4, 6.8 Hz, 1H, H-1), 2.71 (m, 1H, H-3), 2.13 (m, 1H, H-2β), 2.07–2.03 (ddd, *J* = 12.4, 10.3, 3.2 HZ, 1H, H-2α), 2.00 (ddt, *J* = 12.3, 9.3, 5.2 Hz, 1H, H-8), 1.84 (m, 1H, H-7α), 1.78–1.62 (m, 3H, H-5β, H-4β, H-6α), 1.57–1.46 (m, 3H, H-5α, H-6β, H-7β), 1.36 (d, *J* = 13.0 Hz, 1H, H-4α), 1.17 (d, *J* = 7.3 Hz, 3H, H-9), 1.12 (s, 3H,H-10).^13^C NMR (151 MHz, CDCl_3_) δ 175.5 (C-11).56, 173.76 (C-12), 85.60 (C-8a), 52.15(C-13), 42.69(C-1), 42.24 (C-4), 38.92(C-4a), 37.36(C-5), 35.25(C-3), 34.33(C-8), 30.41(C-2), 28.17(C-7), 24.81 (C-10), 16.45 (C-6), 15.77 (C-9). HRMS(EI^+^) calcd for C_15_H_22_O_4_ 266.1518. Found (MH^+^) 266.1510.

#### 4.3.5. 4a-Hydroxy-4-(Methoxycarbonyl)-1,2,3,4,4a,7,8,8a-Octahydronaphthalene-2-Carboxylic Acid (**2e**)

Following the general procedure, a solution of 300 mg (3.0 mmol) of cyclohexenone **1e** in anh. THF was treated with 0.68 mg (3.0 mmol) of LDA. To the resulting enolate was added methyl acrylate (6.6 mmol) to afford 242 mg (31%) of **2e** as a white solid. Mp 113–114 °C. IR (KBr): 3513.6, 3022.1, 2933.8, 2870.8, 1709.3, 1436 cm^−1^. ^1^H NMR (600 MHz, CDCl3) δ 5.85 (ddd, *J* = 9.7, 4.1, 2.7 Hz, 1H, H-7), 5.70 (d, *J* = 9.7, 1.8 Hz, 1H, H-8), 3.76 (s, 3H, OMe), 2.51 (tt, *J* = 12.5, 4.0 Hz, 1H, H-3), 2.43 (dd, *J* = 12.9, 3.9 Hz, 1H, H-1), 2.19 (q, *J* = 12.9 Hz, 1H, 2ax), 2.19–2.05 (m, 3H, H-2eq, H-6α, H-6β), 1.89 (q, *J* = 12.6 Hz, 1H, H-4ax), 1.82 (m, 1H, H-5β), 1.74 (bd, *J* = 12.6 Hz, 1H, H-4eq), 1.45 (m, 2H, H-4a, H-5α). ^13^C NMR (151 MHz, CDCl_3_) δ 180.34 (C-10), 175.69 (C-9), 131.44 (C-7), 129.96 (C-8), 66.93 (C-8a), 51.99 (OMe), 49.74 (C-1), 41.94 (C-4a), 41.82 (C-3), 29.49 (C-4), 27.23 (C-2), 25.99 (C-6), 23.64 (C-5). HRMS(EI^+^) calcd for C_14_H_20_O_5_ 256.1311. Found (MH^+^) 256.1306.

#### 4.3.6. Dimethyl 4a-Hydroxyoctahydro-1H-Spiro [Naphthalene-2,2′-[1,3]Dioxolane]-5,7-Dicarboxylate (**2f** and **2f′**)

Following the general procedure, a solution of 300 mg (1.0 mmol) of 1,4-cyclohexanodione monoethylenketal **1f** in anh. THF was treated with 400 mg (1.0 mmol) of LDA. To the resulting enolate was added methyl acrylate (3.9 mmol) to afford 152 mg (51%) of **2f** as a white solid. Major diastereoisomer **2f**. Mp 135-136 °C. IR (KBr): 3514.6, 2953.4, 2884.7, 1730.8 cm^−1^. ^1^H NMR (600 MHz, CDCl_3_) δ 3.98–3.89 (m, 4H, H-6’), 3.73 (s, OMe), 3.72 (s, 1H, OMe), 3.20 (d, *J* = 1.68 Hz, 1H, -OH), 2.78 (m, 1H, H-3), 2.62 (dd, *J* = 11.9, 4.9 Hz, 1H, H-1), 2.15–2.06 (m, 2H, H-2ax, H-2eq), 2.06–1.96 (m, 2H, H-5eq), 1.86 (m, 1H, H-4eq), 1.75 (m, 1H, H-4ax), 1.79-1.71 (m, 2H, H-4ax, H-8eq), 1.65-1.49 (m, 4H, H-4a, H-7eq, H-7ax, H-5eq), 1.42 (m, 1H, H-8ax). ^13^C NMR (151 MHz, CDCl_3_) δ 176.9 (C-9), 175.0 (C-10), 108.74 (C-6), 68.7 (C8a), 64.31 (C-6′), 64.18 (C-7′), 51.9 (OMe), 51.78 (OMe), 47.6 (C-1), 38.1 (C-3), 36.6 (C-8), 34.7 (C-7), 30.2 (C-5), 28.4 (C-4), 26.2 (C-2).

Minor diastereoisomer **2f′**. ^1^H NMR (600 MHz, CDCl_3_) δ 3.72 (s, OMe), 3.68 (s, OMe), 3.22 (d, *J* = 1.68 Hz, 1H, OH), 2.43 (tt, *J* = 12.4, 3.83 Hz, 1H, H-3), 2.33 (dd, *J* = 12.90, 3.81 Hz, 1H, H-1), 2.09 (m, 1H, H-2eq), 2.05-1.96 (m, 2H, H-2ax, H-5eq), 1.90-1.86 (m, 1H, H-8eq), 1.77 (m, 1H, H-4eq), 1.69-1.49 (m, 5H, H-4a, H-4ax, H-5ax, H-7eq, H-7ax), H-6’), 1.42 (m, 1H, H-8ax). ^13^C NMR (151 MHz, CDCl_3_) δ 176.2 (C-9), 174.8 (C-10), 108.8 (C-6), 68.3 (C8a), 64.3 (C-6’), 64.2 (C-7’), 51.9 (OMe), 51.8 (OMe), 50.49 (C-1), 41.9 (C-3), 40.9 (C-4a), 36.58 (C-8), 34.5 (C-7), 30.19 (C-5), 29.9 (C-4), 27.7 (C-2). HRMS(EI^+^) calcd for C_16_H_24_O_7_ 328.1522. Found (MH^+^) 328.1534.

#### 4.3.7. Methyl 5-Isopropyl-8-Methyl-10-Oxooctahydro-2H-4a,2-(Epoxyme†Hano)naph†Halene-4-Carboxylate (Mixture of **2g**, **2g′** and **2g″**)

Following the general procedure, a solution of 300 mg (1.9 mmol) of menthone **1g** in anh. THF was treated with 0.416 mg (1.94 mmol) of LDA. To the resulting enolate was added methyl acrylate (3.8 mmol) to afford 64 mg (11%) of a mixture **2g**, **2g′** and **2g″** as a yellowish syrup. IR (KBr): 2954.5, 2872.9, 1736.5, 1437.9. ^1^H NMR (600 MHz, CDCl_3_) δ 3.72 and 3.68 (s, 3xOMe) **2g**, **2g′** and **2g″**, 3.35 (dd, *J* = 10.7, 5.2 Hz, 1H, H-1) **2g**, 3.16 (dd, *J* = 10.7, 5.8 Hz, 1H, H-1) **2g′**, 2.73–2.66 (m, 3xH-3) **2g**, **2g′** and **2g″**, 2.60 (dd, *J* = 10.8, 4.8 Hz, H-1) **2g″**. ^13^C NMR (151 MHz, CDCl_3_) δ 177.0-173.0 (6xC = O), 88.5-86.0 (3xC-8a), 53.0-49.0 (3xOMe), 47.78, 47.55, 47.12, 44.88, 41.88, 38.43, 36.41, 35.04, 34.47, 33.69, 30.98, 30.09, 29.95, 29.76, 29.52, 29.13, 27.07, 26.49, 26.31, 25.65, 24.87, 24.46, 23.48, 21.51, 21.31, 19.72, 19.55, 18.52. HRMS(EI^+^) calcd for C_17_H_26_O_4_ 294.1831. Found (MH^+^) 294.1835.

#### 4.3.8. Methyl 8-Methyl-10-Oxo-5-(Propan-2-Ylidene)Octahydro-2H-4a,2-(Epoxymethano)Naphthalene-4-Carboxylate (**2h**)

Following the general procedure, a solution of 100 mg (0.65 mmol) of pulegone **1h** in anh. THF was treated with 0.140 mg (0.65 mmol) of LDA. To the resulting enolate was added methyl acrylate (1.3 mmol) to afford 97 mg (51%) of **2h** as a yellowish syrup. [α]_D_^25^ = −74.0° (c 0.50, CH_2_Cl_2_). IR (KBr): 3462.1, 2953.8, 2928.2, 2871.2, 1738.0 1712.4, 1620.7 cm^−1^. ^1^H NMR (600 MHz, CDCl_3_) δ 3.62 (s, 1H, OMe), 3.31 (dd, *J* = 10.6, 6.0 Hz, 1H, H-1), 2.77–2.71 (m, 1H, H-3 y H-7), 2.34 (dddd, *J* = 13.4, 5.9, 2.9, 2.7 Hz, 1H, H-2β), 2.20 (dddd, *J* = 12.8, 10.2, 4.8, 2.9 Hz, 1H, H-4β), 2.01 (ddd, *J* = 13.6, 10.6, 3.1 Hz, 1H, H-2α), 1.96 (s, 3H, Me-12), 1.91 (m, 1H, H-7′), 1.77–1.70 (s/m, 3H/1H, Me-11/H-6), 1.59–1.46 (ddd, *J* = 11.1, 10.2, 6.8 Hz, 1H, H-4a), 1.50 (m, 1H, H-5), 1.32 (ddd, *J* = 13.2, 7.8, 1.5 Hz, 1H, H-4α), 1.1), 1.32 (ddd, *J* = 13.2, 7.8, 1.5 Hz, 1H, H-0–1.02 (m, 1H, H-6), 0.97 (d, *J* = 6.2 Hz, 3H, H-14).^13^C NMR (151 MHz, CDCl3) δ 174.8 (C-10), 172.9 (C-9), 128.7 (C-8), 126.4 (C-13), 87.9 (C-8a), 51.9 (C-15), 49.0 (C-4a), 41.2 (C-1), 34.09 (C-5), 34.06 (C-6 and C-3), 29.3 (C-4), 28.3 (C-2), 28.3 (C-7), 23.9 (C-11), 22.5 (C-12),19.7 (C-14). HRMS(EI^+^) calcd for C_17_H_24_O_4_ 292.1675 Found (MH^+^) 292.1669. 

#### 4.3.9. Methyl-8-Methyl-10-Oxo-5-(Prop-1-En-2-yl)1,3,4,5,6,8a-Hexahydro-2H-4a,2-(Epoxymethano)Naphthalene-4-Carboxylate (**2i**)

Following the general procedure, a solution of 100 mg (0.66 mmol) of carvone **1i** in anh. THF was treated with 0.142 mg (0.6 mmol) of LDA. To the resulting enolate was added methyl acrylate (1.32 mmol) to afford 72 mg (37%) of **2i** as a white solid. Mp: 144–145 °C. [α]_D_^25^ = −65.3° (c 0.70, CH_2_Cl_2_). IR (KBr): 3447.5, 2951.6, 1732.6, 1436.6 cm^−1^. ^1^H NMR (600 MHz, CDCl_3_) δ 5.87 (bd, *J* = 1.46 Hz 1H, H-7), 4.87 (m, 1H, H-11a), 4.80 (m, 1H, H-11b), 3.73 (s, 3H, OMe), 2.74-2.78 (m, 2H, H-1, H-3), 2.23–2.12 (m, 3H, H-5, H-2α, 2β), 2.10–1.97 (m, 3H, H-4α, H-6α, H-6β), 1.83 (m, 1H, H-4a), 1.77 (quint, *J* = 1.33 Hz, 3H, H-9), 1.67 (bq, *J* = 0.8 Hz, 3H, H-12), 1.59 (m, 1H, H-4β). ^13^C NMR (151 MHz, CDCl3) δ 175.0 (C-15), 174.5 (C-13), 144.7 (C-10), 130.9 (C-7), 130.7 (C-8), 114.1 (C-11), 82.50 (C-8a), 52.22 (C-14), 47.46 (C-1), 44.52 (C-5), 39.68 (C-4a), 34.34 (C-3), 31.08 (C-6), 29.68 (C-4), 29.29 (C-2), 18.81 (C-12), 18.29 (C-9). HRMS(EI^+^) calcd for C_17_H_22_O_4_ 290.1518. Found (MH^+^) 290.1519.

#### 4.3.10. Dimethyl 4a-Hydroxytetradecahydrophenanthrene-2,4-Dicarboxylate (**2j**)

Following the general procedure, a solution of 100 mg (0.65 mmol) of trans-decahydro-1-naphthalenone **1j** in anh. THF was treated with 0.140 mg (0.65 mmol) of LDA. To the resulting enolate was added methyl acrylate (1.31 mmol) to afford 124 mg (58.2%) of **2j** as colorless crystals. 

Mp: 39–40 °C. IR (KBr): 3480.1, 2929.2, 2854.2, 1734.9, 1434.9 cm^−1^. ^1^H NMR (600 MHz, CDCl_3_) δ 3.73 (s, 3H, H-12), 3.69 (s, 3H, H-14), 3.28 (S, 1H, OH), 3.09 (dd, *J* = 4.7, 2.8 Hz, 1H, H-4), 2.65 (tt, *J* = 12.8, 4.0 Hz, 1H, H-2), 2.24–2.18 (m, 2H, H-3eq, H-10a), 2.12 (tt, *J* = 13.5, 4.6 Hz, 1H, H-10ax), 1.90 (q, *J* = 13.2 Hz, 1H, H-1ax), 1.84–1.75 (m, 2H, H-3ax, H-6eq), 1.73 (m, 1H, H-1eq), 1.67–1.52 (m, 4H, H-7eq, H-8eq, H-5eq, H-4b), 1.46 (t, *J* = 9.0 Hz, 1H, H-8a), 1.37 (dt, *J* = 13.2, 3.3 Hz, 1H, H-9eq), 1.30–1.17 (m, 5H, H-5ax, H-6ax, H-8ax, H-9ax, H-10eq), 1.04–0.94 (m, 1H, H-7ax). ^13^C NMR (151 MHz, CDCl_3_) δ 175.82 (C-11), 175.68 (C-13), 73.16 (C-4a), 51.89 (C-12), 51.73 (C-14), 45.13 (C-4), 41.68 (C-8a), 39.25 (C-2), 38.67 (C-10a), 36.84 (C-4b), 34.75 (C-7), 30.97 (C-1), 28.39 (C-9), 27.77 (C-3), 26.85 (C-6), 26.18(C-10), 26.02 (C-8), 24.36 (C-5). HRMS(EI^+^) calcd for C_18_H_28_O_5_ 324.1937 Found (MH^+^) 324.1934.

#### 4.3.11. Dimethyl 3a-Hydroxyoctahydro-1H-Indene-4-6-Carboxylate (**2k**)

Following the general procedure, a solution of 100 mg (1.1 mmol) of cyclopentanone **1k** in anh. THF was treated with 0.254 mg (1.18 mmol) of LDA. To the resulting enolate was added methyl acrylate (3.5 mmol) to afford 75 mg (75%) of **2k** as a white solid. Mp 57–58 °C. IR (KBr): 3507.2, 2953.1, 2879.6, 1732.8 cm^−1^. ^1^H NMR (600 MHz, CDCl_3_) δ 4.03 (s, 1H, OH), 3.75 (s, 3H, H-11), 3.70 (s, 3H, H-10), 2.57 (m, 1H, H-3), 2.52 (m, 1H, H-1), 2.19 (m, 1H, H-4a), 2.07–1.98 (m, 2H, H-2α, H-2β), 1.94 (td, 1H, H-4β), 1.86–1.72 (m, 5H, H-5β, H-6α, H-6β, H-7β, H-4α), 1.61 (m, 1H, H-7α), 1.48 (m, 1H, H-5α). ^13^C NMR (151 MHz, CDCl_3_) δ 176.1 (C-9), 175.4 (C-8), 77.0 (C-7a), 52.0 (C-11), 51.8 (C-10), 44.9 (C-3), 43.9 (C-4a), 38.2 (C-6), 37.6 (C-1), 27.4 (C-2), 26.1 (C-4), 25.5 (C-5), 19.5 (C-7). HRMS(EI^+^) calcd for C_13_H_20_O_5_ 256.1311. Found (MH^+^) 256.1312.

#### 4.3.12. Dimethyl 3a-Hydroxy-3-Methyloctahydro-1H-Indene-4,6-Dicarboxylate (**2l**)

Following the general procedure, a solution of 100 mg (1.0 mmol) of 2-methylcyclopentanone **1l** in anh. THF was treated with 0.220 mg (1.0 mmol) of LDA. To the resulting enolate was added methyl acrylate (2.0 mmol) to afford 250 mg (91%) of **2l** as a colorless syrup. IR (KBr): 3526.7, 2953.0, 2873.5, 1732.0 cm^−1^. ^1^H NMR (600 MHz, CDCl_3_) δ 3.75 (s, 3H, H-10), 3.68 (s, 3H, H-11), 2.91 (dd, *J* = 5.4, 4.3 Hz, 1H, H-1), 2.70 (s, 1H, OH), 2.66 (tt, *J* = 11.9, 4.1 Hz, 1H, H-3), 2.30–2.24 (m, 1H, H-4a), 2.18–2.00 (m, 3H, H-6β, H-7, H-2β), 1.98–1.85 (m, 3H, H-2α, H-5β, H-4β), 1.43 (m, 1H, H-5α), 1.34 (q, *J* = 13.3 Hz, 1H, H-4α), 1.24 (m, 1H, H-6α), 0.96 (d, *J* = 6.8 Hz, 3H, H-12).^13^C NMR (151 MHz, CDCl_3_) δ 171.7 (C-8, C-9), 79.8 (C7a), 51.92 (C-10), 51.75 (C-11), 44.96 (C1), 44.64 (C-4a), 38.01 (C-3), 37.63 (C-7), 32.86 (C-4), 29.34 (C-5), 28.31 (C-6), 28.01 (C-2), 12.81 (C-12). HRMS(EI^+^) calcd for C_14_H_22_O_5_ 270.1472 Found (MH^+^) 270.1467. 

## Figures and Tables

**Table 1:** Structures of decalin and hydrindane derivatives obtained in higher yields among 8 possible diastereoisomers that could be generated by the MIMIRC reaction between cycloalkanone enolates and methyl acrylate.

Entry ^a^	Ketone	Product	Yield (%) ^b^	Entry ^a^	Ketone	Product	Yield (%) ^b^
1	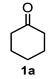	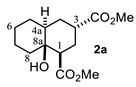	43	7	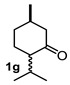	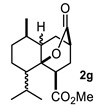	11
2	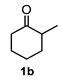	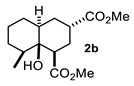	42	8	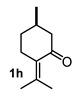	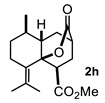	51
3	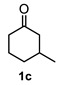	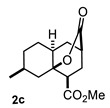	77	9	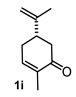	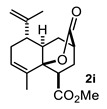	37
4	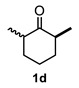	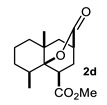	52	10	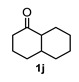	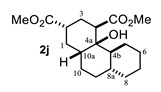	58
5	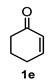	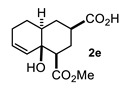	31	11	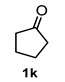	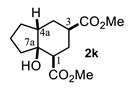	75
6	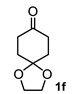	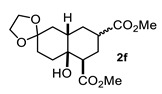	51	12	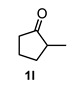	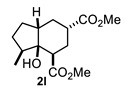	91

^a^ All reactions were performed in anhydrous THF at −78 °C under N_2_ atmosphere. ^b^ Chemical yields are reported after column chromatography separation.

**Table 2:** Relative free energies (ΔG, kcal/mol, 1 atm, 25 °C) ^a^ obtained for the 4–5 most stable conformers of lithium alkoxides **I–VIII**.

Conformer	I ^b^	II ^b^	III	IV	V ^b^	VI ^b^	VII	VIII
**a**	1.85	3.41	18.39	0.91	3.28	3.11	5.68	0.00
**b**	2.77	4.52	18.48	1.39	4.28	3.87	7.43	0.97
**c**	9.31	10.00	18.51	9.22	11.38	10.01	11.85	7.51
**d**	10.89	11.16	19.97	9.42	12.11	10.06	13.23	8.94
**e**						10.33		

^a^ Relative to the most stable conformer of the most stable diastereoisomer (**VIII**, **2n**). ^b^ For *cis*-decalins, only energies obtained for the most stable conformation of the bicycle are shown.

**Table 3:** Populations (%) of the most stable geometries for systems **I**–**VIII**, obtained from the relative free energies (ΔG, 25 °C) and the Boltzmann distribution law.

Geometry	ΔG (kcal/mol)	Population (%)	Diast. Ratio (%) ^a^
**VIIIa**	0.00	64.1	76.6 (VIII, 2m)
**VIIIb**	0.97	12.5	
**IVa**	0.91	13.9	20.0 (IV, 2a)
**IVb**	1.39	6.1	
**Ia**	1.85	2.8	3.4 (I, 2n)
**Ib**	2.77	0.6	

**^a^** Obtained for each system as the sum of the contributions of the two most significant conformers.

## Data Availability

All relevant spectroscopic data, including X-ray data, are provided in the Supplementary Material.

## Supplementary Materials

The following supporting information can be downloaded at: https://www.mdpi.com/article/10.3390/molecules27092810/s1. Experimental ^1^H, ^13^C NMR and HRMS spectra for synthesized compounds are available. Representative simulated ^1^H NMR spectra and parameters are also provided. Crystallographic data for **2c**, **2d**, and **2j**.

## Sample Availability

Samples of the compounds are not available from authors.
