# Supplementary material for: Structural Analysis of the Michael-Michael Ring Closure (MIMIRC) Reaction Products
Source: Molecules. 2022 Apr 28;27(9):2810. doi: 10.3390/molecules27092810 (PMC9104055; doi:10.3390/molecules27092810)
Supplement: Supplementary file 1 [file molecules-27-02810-s001.zip › molecules-1677285-supplementary.pdf]

SUPPLEMENTARY MATERIALS

Structural analysis of the Michael-Michael Ring Closure (MIMIRC) reaction products

Mabel M. Montenegro-Sustaita, Hugo A. Jiménez-Vázquez, Elena Vargas-Díaz, J. Enrique Herbert-Pucheta and L. Gerardo Zepeda-Vallejo*

Departamento de Química Orgánica, Escuela Nacional de Ciencias Biológicas, Instituto Politécnico Nacional, Prolongación de Carpio y Plan de Ayala s/n, Col. Santo Tomas. C.P. 11340, Delegación Miguel Hidalgo, Ciudad de México. México.

*Correspondence: lzepeda@ipn.mx

Table of contents

Page

1. X-ray Crystallographic Information and ORTEP structures of **2c**, **2d** and **2j** 2
2. NMR (^{1}H, ^{13}C, 1D nOe) and HRMS spectra 9
3. Tables of ^{1}H NMR parameters expected and calculated for simulated ^{1}H NMR spectra of decalins **2a**, **2e**, **2h**, **2i** and hydrindanes **2k** and **2l** 28
4. Simulated ^{1}H NMR spectra of **2a**, **2e** and **2d** 32

1. X-ray Crystallographic Information for compounds **2c**, **2d** and **2j**

X-ray data collection and refinement of crystal structures, crystal data and refinement details for methyl 7-methyl-10-oxooctahydro-2H-4a,2-(epoxymethano)naphthalene-4-carboxylate **2c**, methyl 5,8a-dimethyl-10-oxooctahydro-2H-4a,2-(epoxymethano)naphthalene-4-carboxylate **2d** and dimethyl 4a-hydroxy-tetra-decahydrophenanthrene-2,4-dicarboxylate **2j** are given in Tables S1, S2 and S3, respectively. Single crystals were obtained by slow evaporation of concentrated solutions of decalins **2c**, **2d** and **2j** (*n*-hexane/EtOAc, 7:3, colorless crystals). These were mounted on glass rods. Crystallographic measurements were carried out on an Oxford Diffraction Xcalibur S single-crystal X-ray diffractometer using an area detector and Mo Kα radiation (λ = 0.71073 Å, graphite monochromator) at r.t. Unit cell parameters were obtained from least-squares refinement. Data collection was carried out at 21 °C using ω scans. Intensities were corrected for Lorentz and polarization effects. Empirical (multi-scan) absorption corrections were applied. Structures were solved with SHELXT and refined with SHELXL, both within the WinGX environment. Anisotropic temperature factors were introduced for all non-hydrogen atoms. Hydrogen atoms were placed in idealized positions and their atomic coordinates refined using unit weights, except for hydrogens on oxygen. For the latter, hydrogen atoms were located from the difference density map and their positions and isotropic temperature factors were refined accordingly. ORTEP plots were drawn with PLATON. The structures have been deposited at the CCDC with deposition numbers 2168061 (**2c**), 2168062 (**2d**), and 2168063 (**2j**). These data can be obtained free of charge via http://www.ccdc.cam.ac.uk/conts/retrieving.html (or from the CCDC, 12 Union Road, Cambridge CB2 1EZ, UK; Fax: +44 1223 336033; E-mail: deposit@ccdc.cam.ac.uk). A summary of the crystallographic information of these structures is shown in Tables S1, S2 y S3.

Table S1. Crystal data and structure refinement for **2c**.

Identification code	0047-lgzv	
Empirical formula	C14 H20 O4	
Formula weight	252.30	
Temperature	292(2) K	
Wavelength	0.71073 Å	
Crystal system	Monoclinic	
Space group	I a	
Unit cell dimensions	a = 13.5825(18) Å	α= 90°.
	b = 8.3639(11) Å	β= 105.101(15)°.
	c = 12.0639(19) Å	γ = 90°.
Volume	1323.2(3) $Å^3$	
Z	4	
Density (calculated)	1.267 Mg/m^3	
Absorption coefficient	0.092 mm^{-1}	
F(000)	544	
Crystal size	0.430 x 0.190 x 0.080 mm^3	
Theta range for data collection	2.889 to 29.356°.	
Index ranges	-18<=h<=16, -11<=k<=5, -15<=l<=15	
Reflections collected	2872	
Independent reflections	1954 [R(int) = 0.0199]	
Completeness to theta = 25.242°	100.0 %	
Absorption correction	Semi-empirical from equivalents	
Max. and min. transmission	0.993 and 0.416	
Refinement method	Full-matrix least-squares on F^2	
Data / restraints / parameters	1954 / 2 / 165	
Goodness-of-fit on F^2	1.029	
Final R indices [I>2sigma(I)]	R1 = 0.0459, wR2 = 0.1049	
R indices (all data)	R1 = 0.0667, wR2 = 0.1217	
Absolute structure parameter	-1.1(10)	
Extinction coefficient	n/a	
Largest diff. peak and hole	0.173 and -0.142 $e.Å^{-3}$	

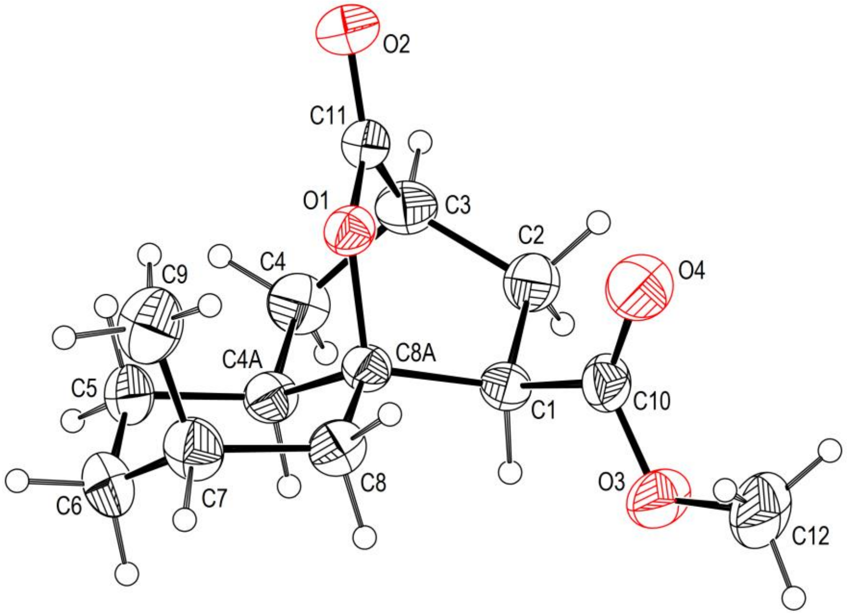

Figure S1. X-Ray diffraction of methyl 7-methyl-10-oxooctahydro-2H-4a,2-(epoxymethano)naphthalene-4-carboxylate **2c**.

Table S2. Crystal data and structure refinement for **2d**.

Identification code	0044_lgzv	
Empirical formula	C15 H22 O4	
Formula weight	266.32	
Temperature	292(2) K	
Wavelength	0.71073 Å	
Crystal system	Monoclinic	
Space group	P 21/c	
Unit cell dimensions	a = 8.0880(3) Å	α= 90°.
	b = 10.6182(3) Å	β= 99.104(3)°.
	c = 16.1597(4) Å	γ = 90°.
Volume	1370.31(7) Å^3	
Z	4	
Density (calculated)	1.291 Mg/m^3	
Absorption coefficient	0.092 mm^{-1}	
F(000)	576	
Crystal size	0.780 x 0.670 x 0.270 mm^3	
Theta range for data collection	3.284 to 32.547°.	
Index ranges	-10<=h<=11, -16<=k<=15, -24<=l<=24	
Reflections collected	14881	
Independent reflections	4538 [R(int) = 0.0179]	
Completeness to theta = 25.242°	99.7 %	
Absorption correction	Semi-empirical from equivalents	
Max. and min. transmission	0.976 and 0.943	
Refinement method	Full-matrix least-squares on F^2	
Data / restraints / parameters	4538 / 0 / 175	
Goodness-of-fit on F^2	1.060	
Final R indices [I>2sigma(I)]	R1 = 0.0519, wR2 = 0.1341	
R indices (all data)	R1 = 0.0681, wR2 = 0.1441	
Extinction coefficient	n/a	
Largest diff. peak and hole	0.315 and -0.150 e.Å^{-3}	

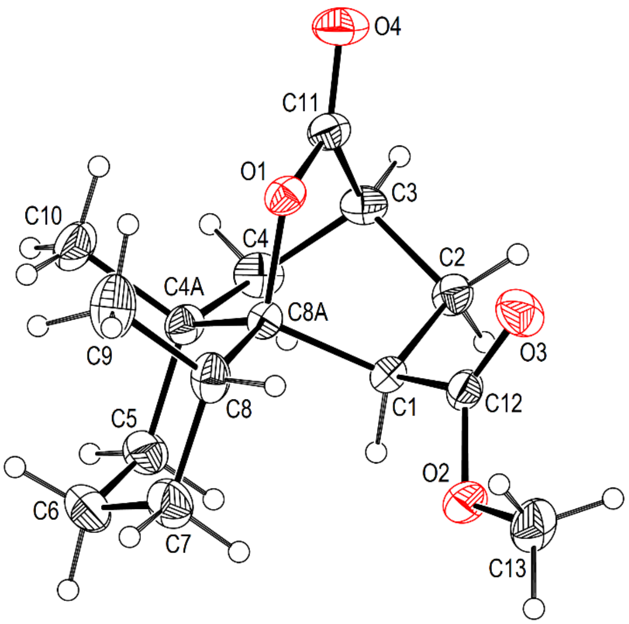

Figure S2. X-Ray diffraction structure of methyl 5,8a-dimethyl-10-oxooctahydro-2H-4a,2-(epoxymethano)naphthalene-4-carboxylate **2d**.

Table S3. Crystal data and structure refinement for **2j**.

Identification code	0056-lgzv	
Empirical formula	C18 H28 O5	
Formula weight	324.40	
Temperature	292(2) K	
Wavelength	0.71073 Å	
Crystal system	Monoclinic	
Space group	C 2/c	
Unit cell dimensions	a = 25.9424(10) Å	α= 90°.
	b = 10.2211(4) Å	β= 99.696(4)°.
	c = 13.0944(4) Å	γ = 90°.
Volume	3422.5(2) Å^3	
Z	8	
Density (calculated)	1.259 Mg/m^3	
Absorption coefficient	0.090 mm^{-1}	
F(000)	1408	
Crystal size	0.600 x 0.550 x 0.100 mm^3	
Theta range for data collection	3.112 to 29.640°.	
Index ranges	-34<=h<=35, -13<=k<=12, -18<=l<=17	
Reflections collected	24220	
Independent reflections	4344 [R(int) = 0.0325]	
Completeness to theta = 25.242°	99.8 %	
Absorption correction	Semi-empirical from equivalents	
Max. and min. transmission	0.991 and 0.609	
Refinement method	Full-matrix least-squares on F^2	
Data / restraints / parameters	4344 / 0 / 214	
Goodness-of-fit on F^2	1.029	
Final R indices [I>2sigma(I)]	R1 = 0.0537, wR2 = 0.1227	
R indices (all data)	R1 = 0.0764, wR2 = 0.1357	
Extinction coefficient	n/a	
Largest diff. peak and hole	0.323 and -0.201 e.Å^{-3}	

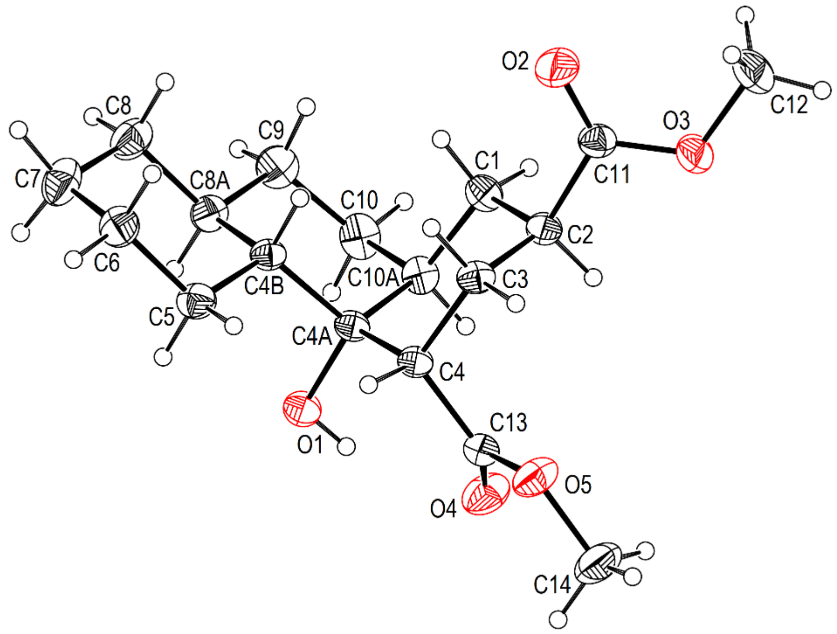

Figure S3. X-Ray diffraction of dimethyl 4a-hydroxy-tetra-decahydrophenanthrene-2,4-dicarboxylate **2j**.

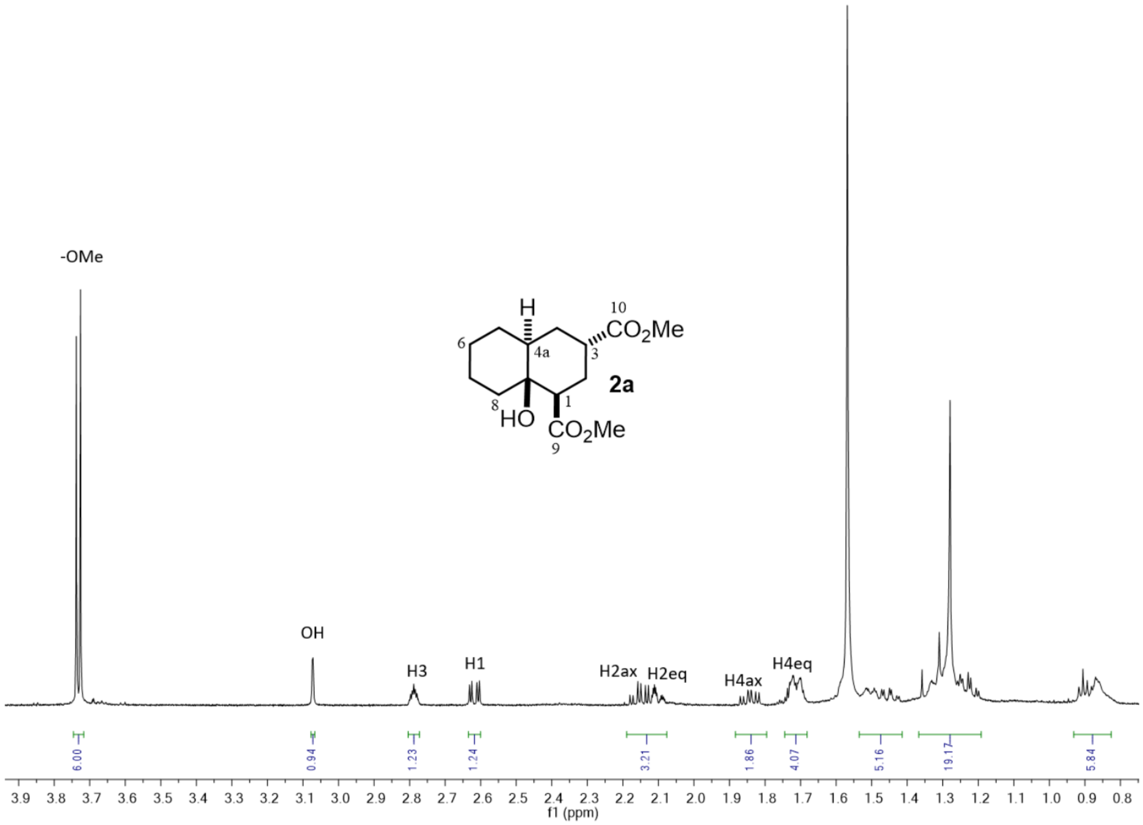

Figure S4. ^{1}H NMR spectrum of decalin **2a** (600 MHz, $CDCl_3$).

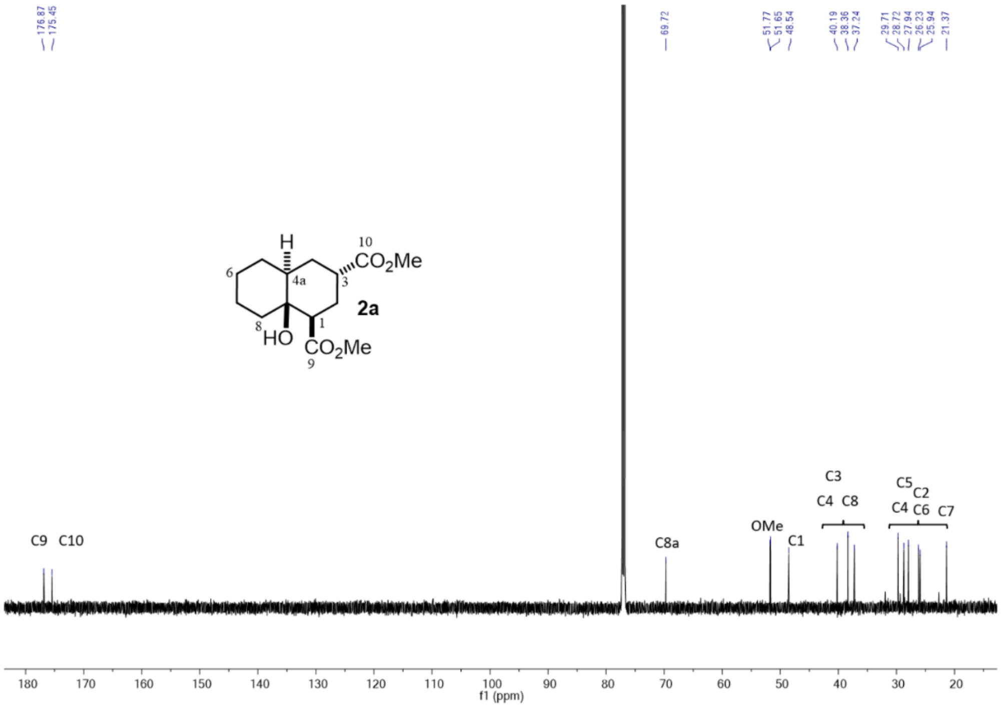

Figure S5. ^{13}C NMR spectrum of decalin **2a** (150 MHz, $CDCl_3$).

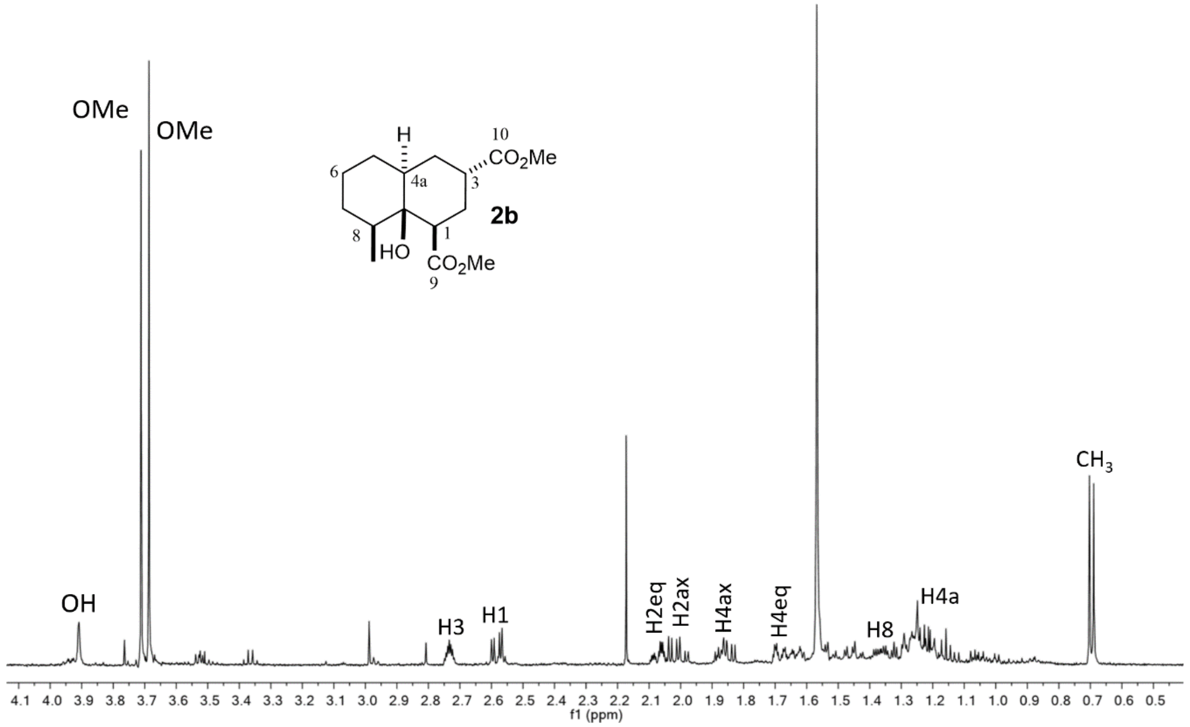

Figure S6. ^{1}H NMR spectrum of decalin **2b** (500 MHz, $CDCl_3$).

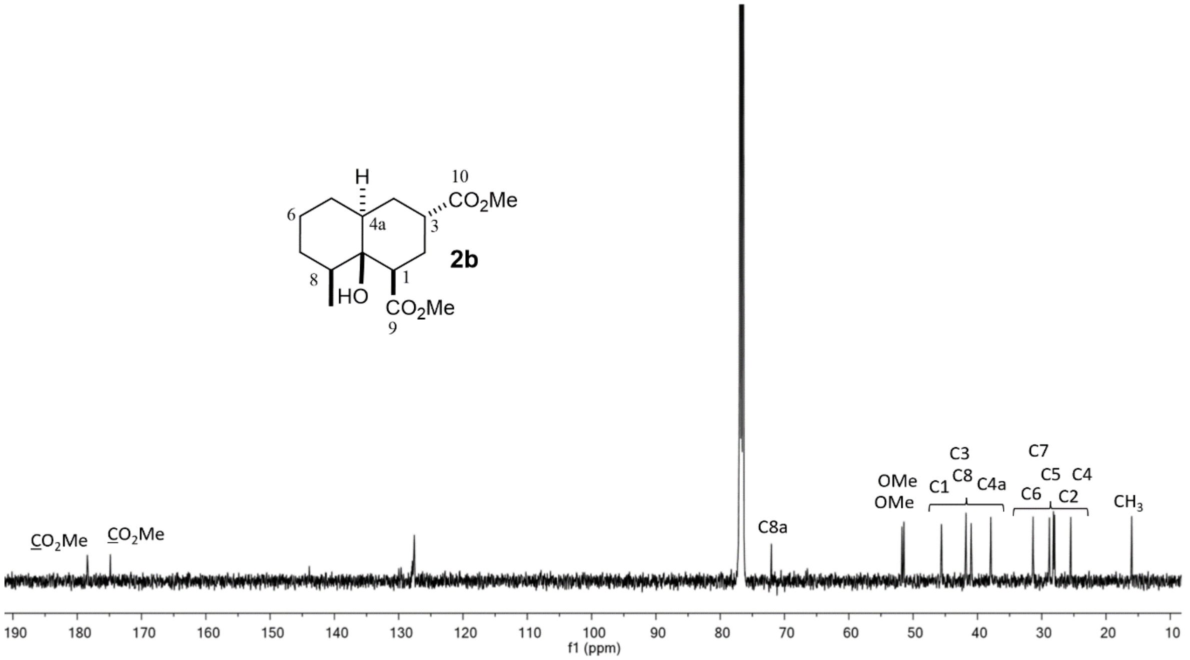

Figure S7. ^{13}C NMR spectrum of decalin **2b** (125 MHz, $CDCl_3$).

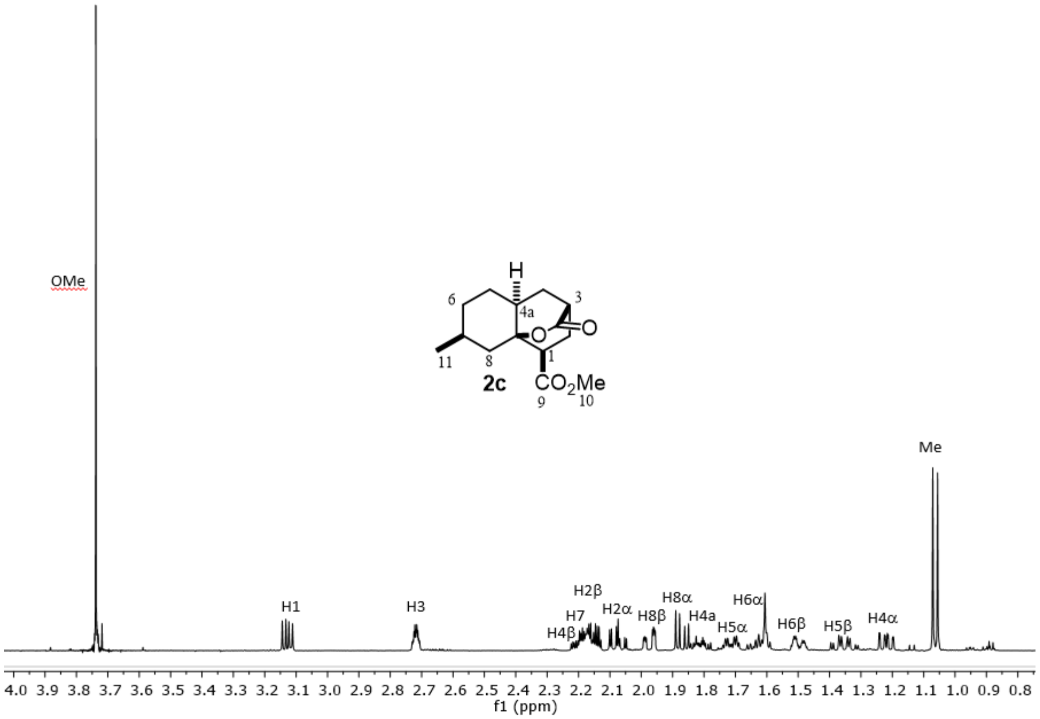

Figure S8. ^{1}H NMR spectrum of decalin **2c** (600 MHz, $CDCl_3$).

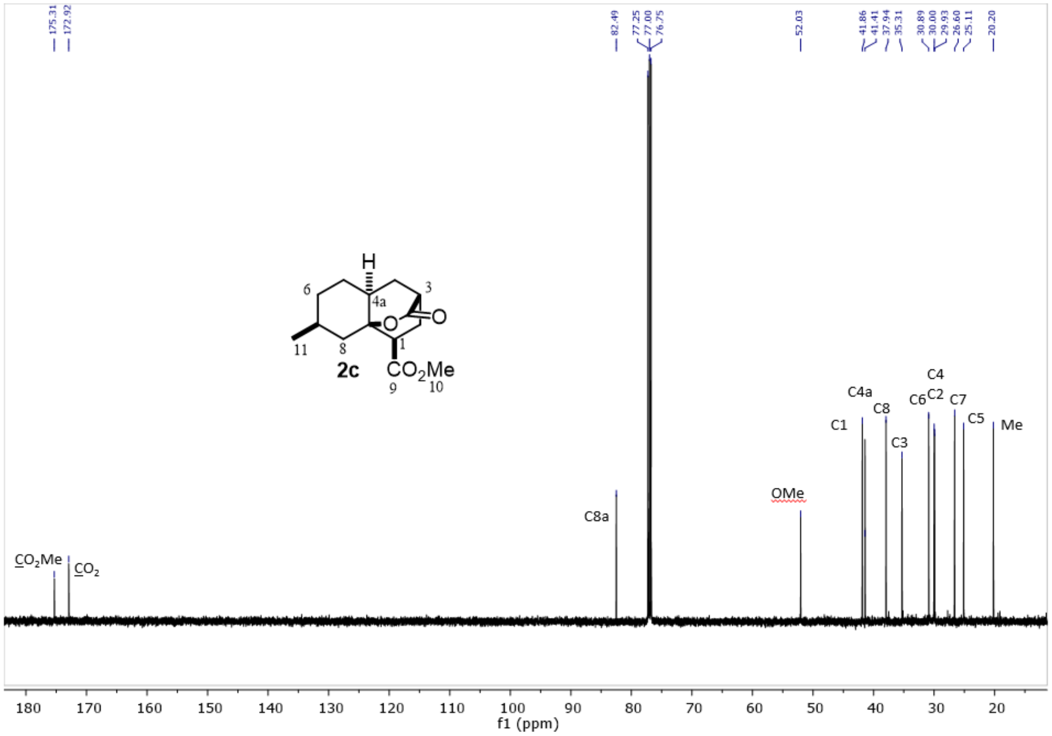

Figure S9. ^{13}C NMR spectrum of decalin **2c** (150 MHz, $CDCl_3$).

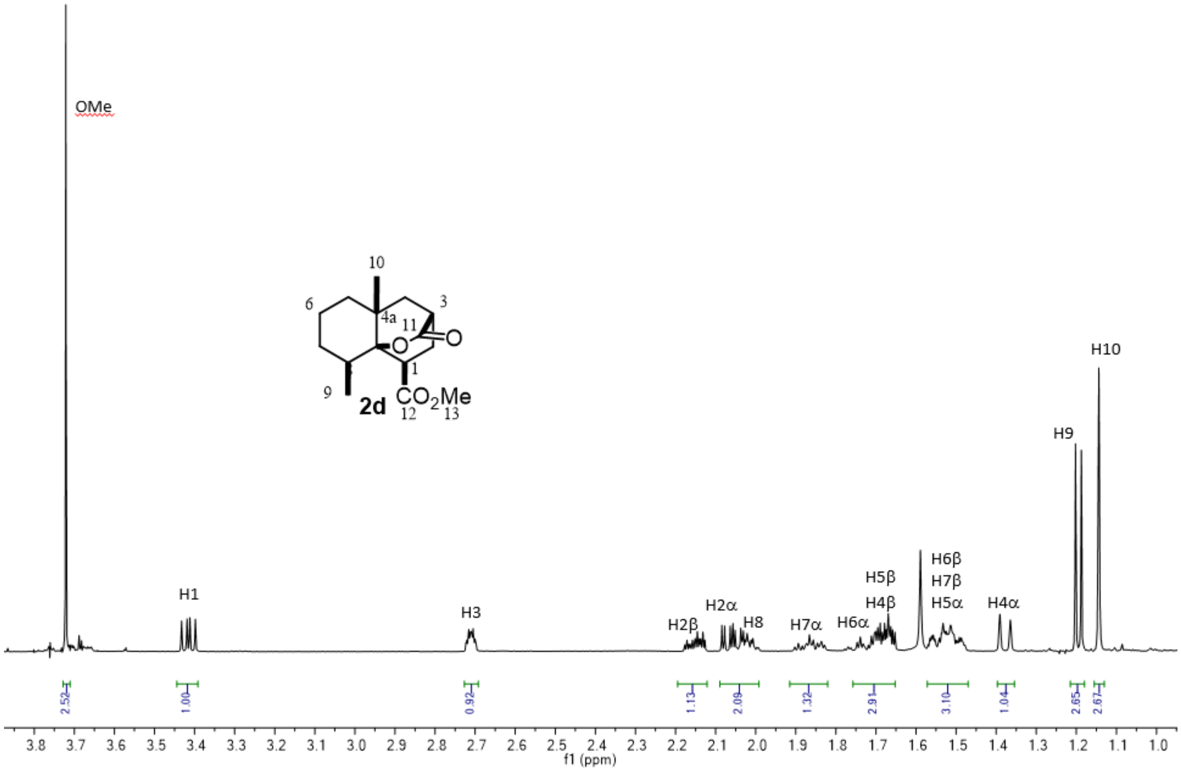

Figure S10. ^{1}H NMR spectrum of decalin **2d** (600 MHz, $CDCl_3$).

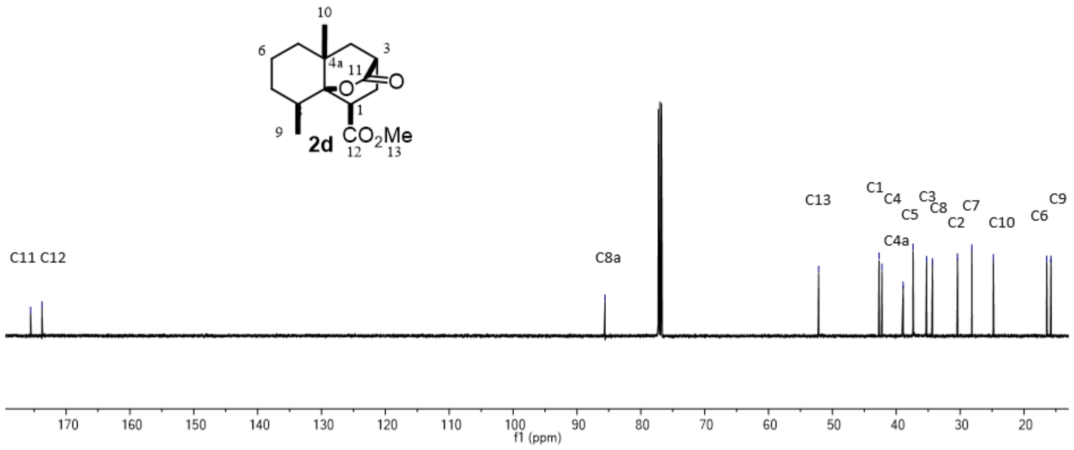

Figure S11. ^{13}C NMR spectrum of decalin **2d** (150 MHz, $CDCl_3$).

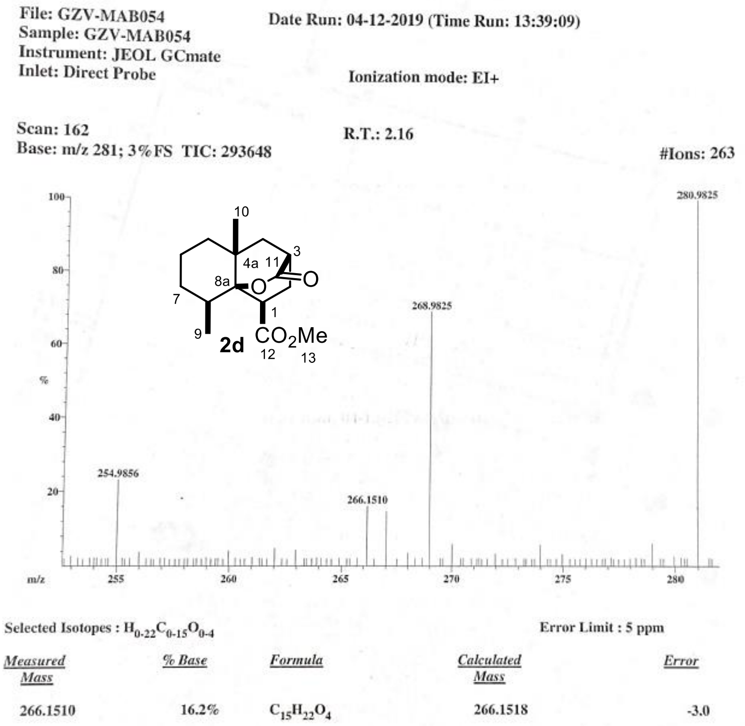

Figure S12. High-resolution mass spectrum of decalin **2d**.

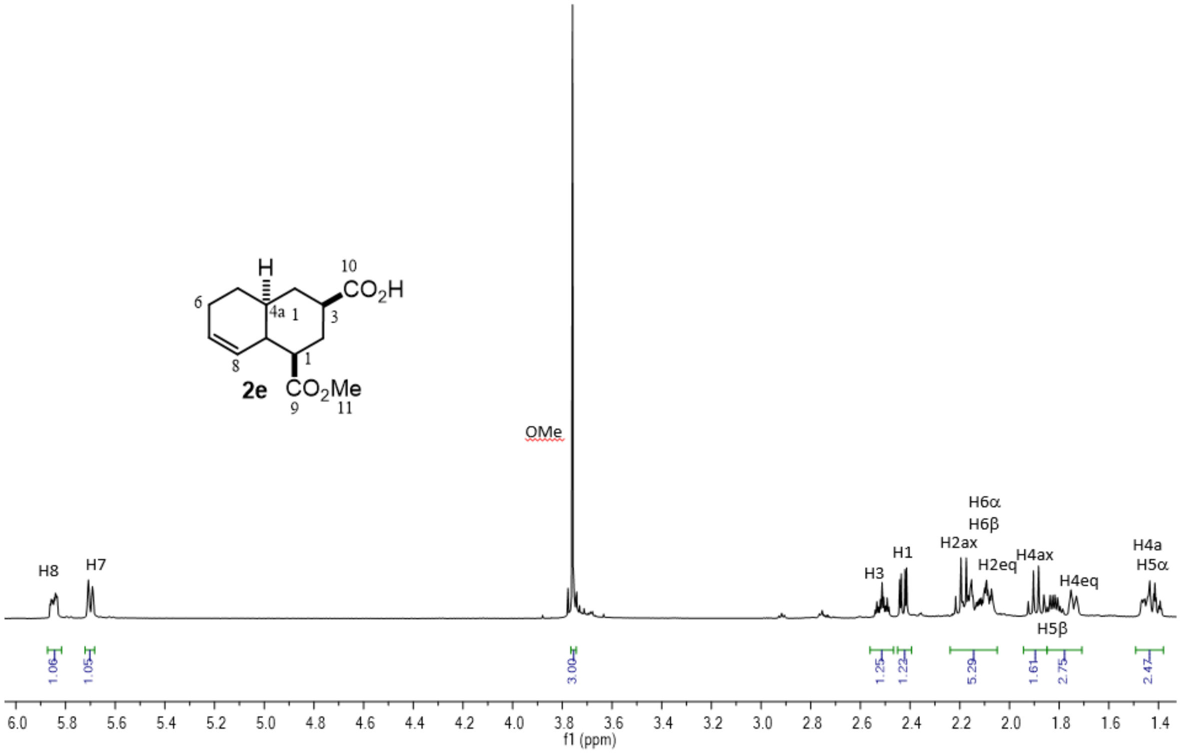

Figure S13. ^{1}H NMR spectrum of decalin **2e** (600 MHz, $CDCl_3$).

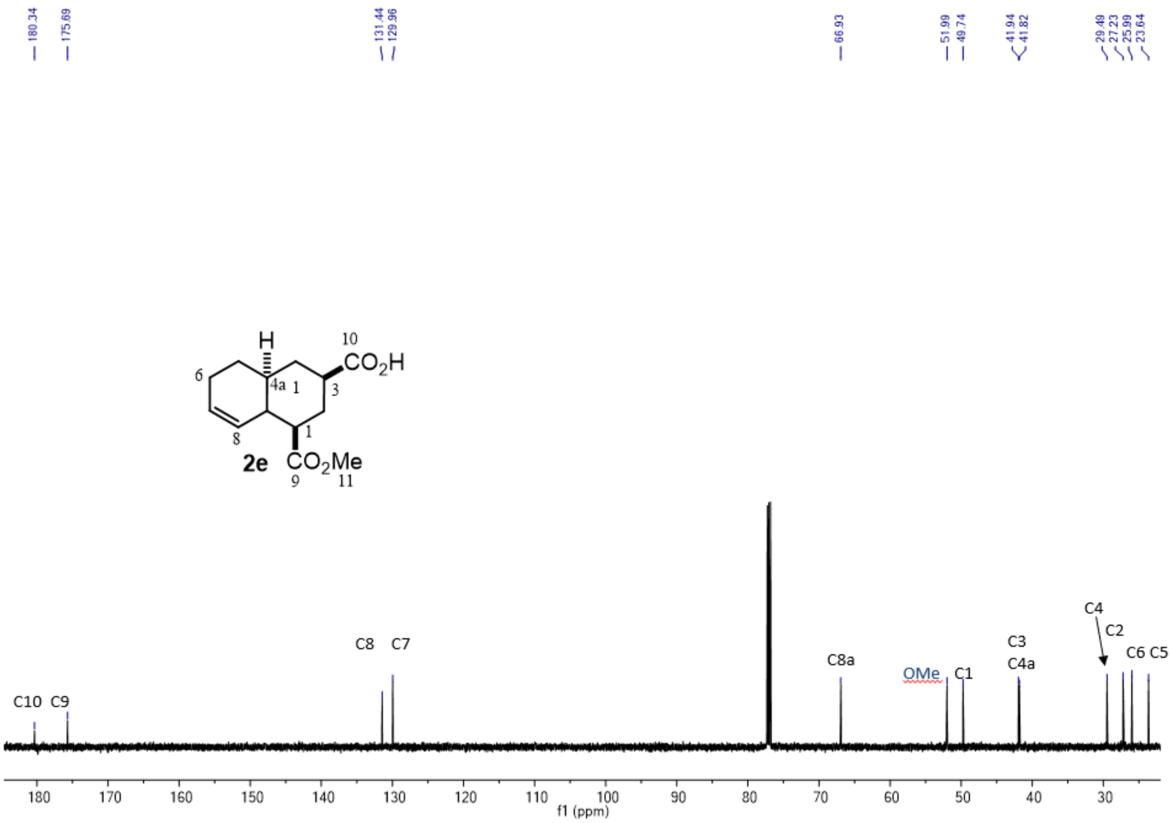

Figure S14. ^{13}C NMR spectrum of decalin **2e** (150 MHz, $CDCl_3$).

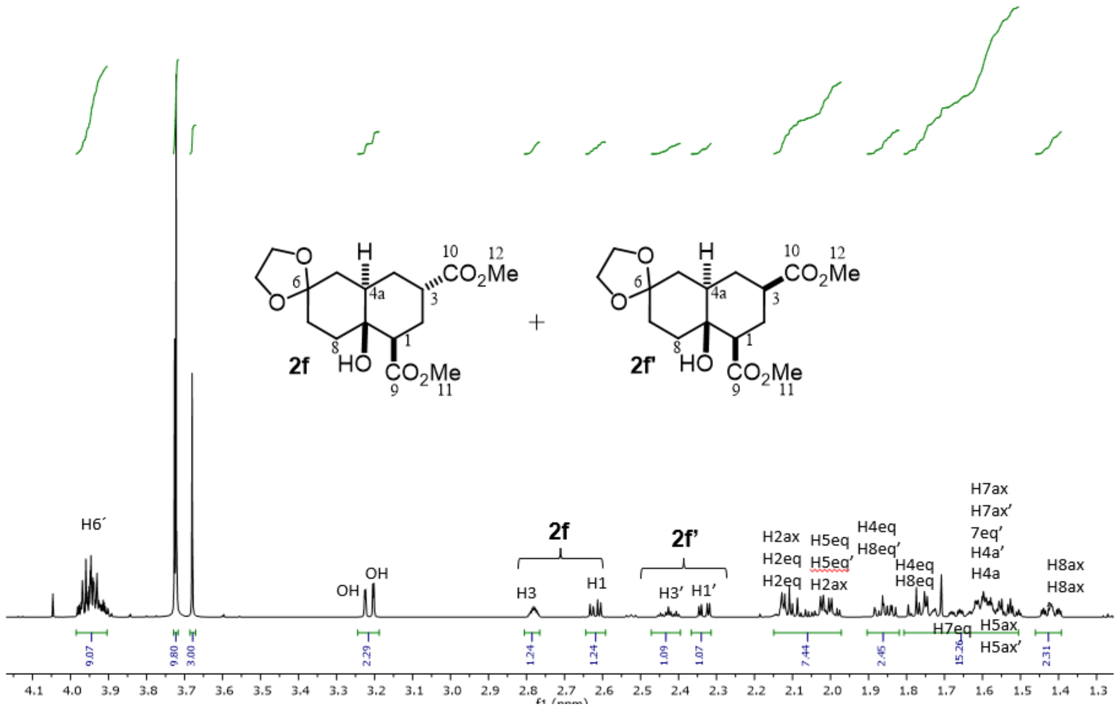

Figure S15. ^{1}H NMR spectrum of the mixture of decalins **2f** and **2f’** (600 MHz, $CDCl_3$).

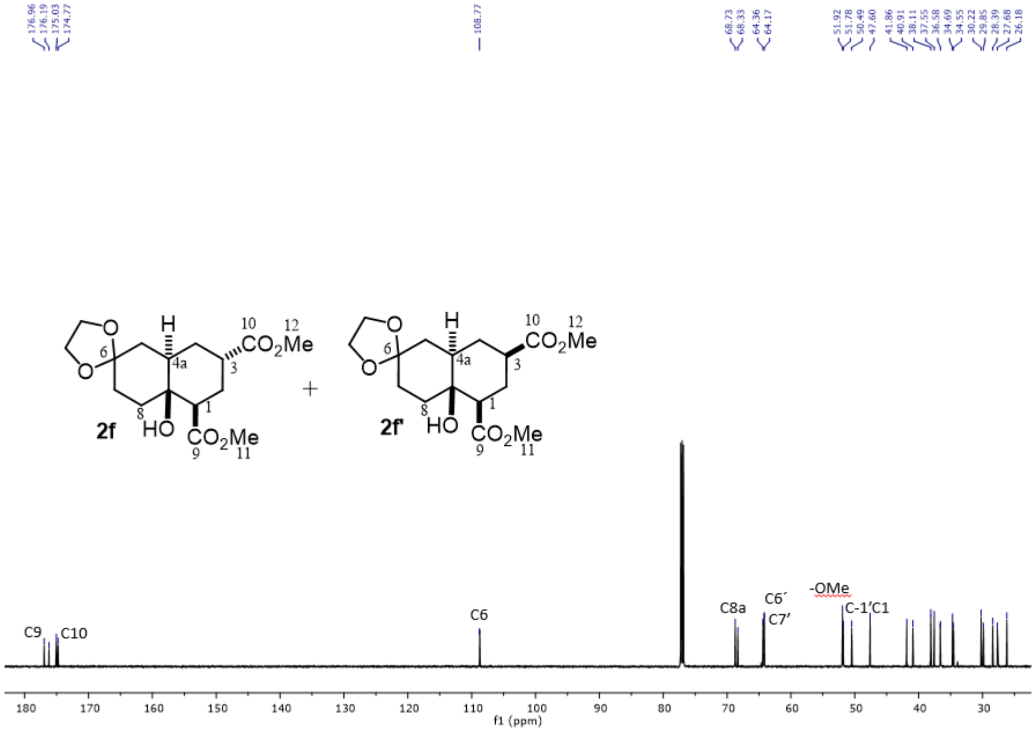

Figure S16. ^{13}C NMR spectrum of decalins **2f** (major) and **f'** (minor) (150 MHz, $CDCl_3$).

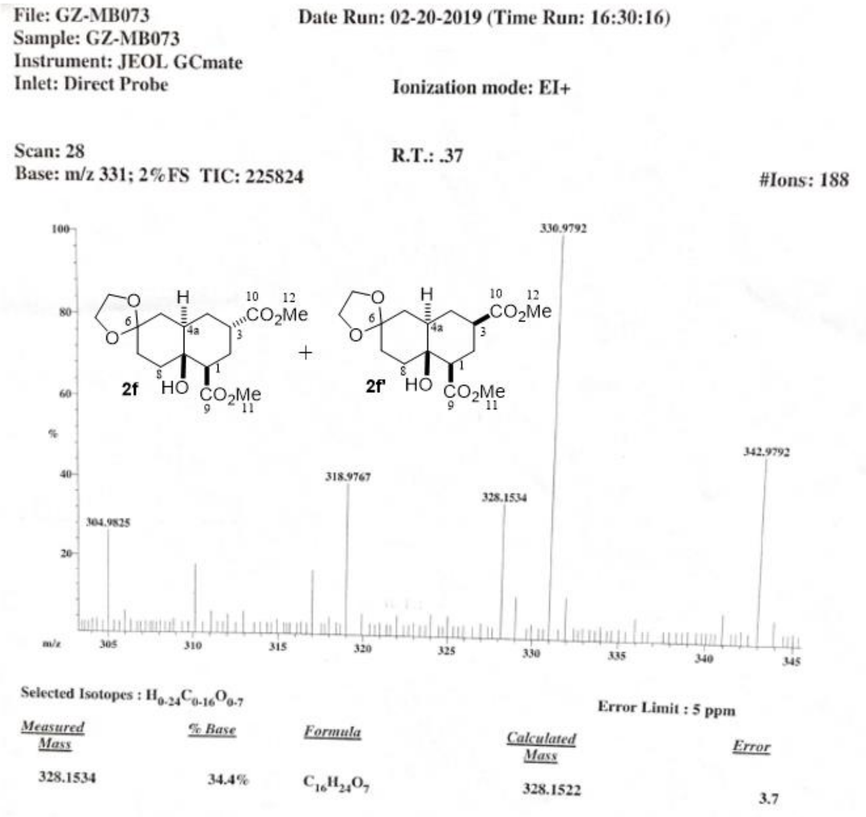

Figure S17. High-resolution mass spectrum of decalins **2f** and **2f'**.

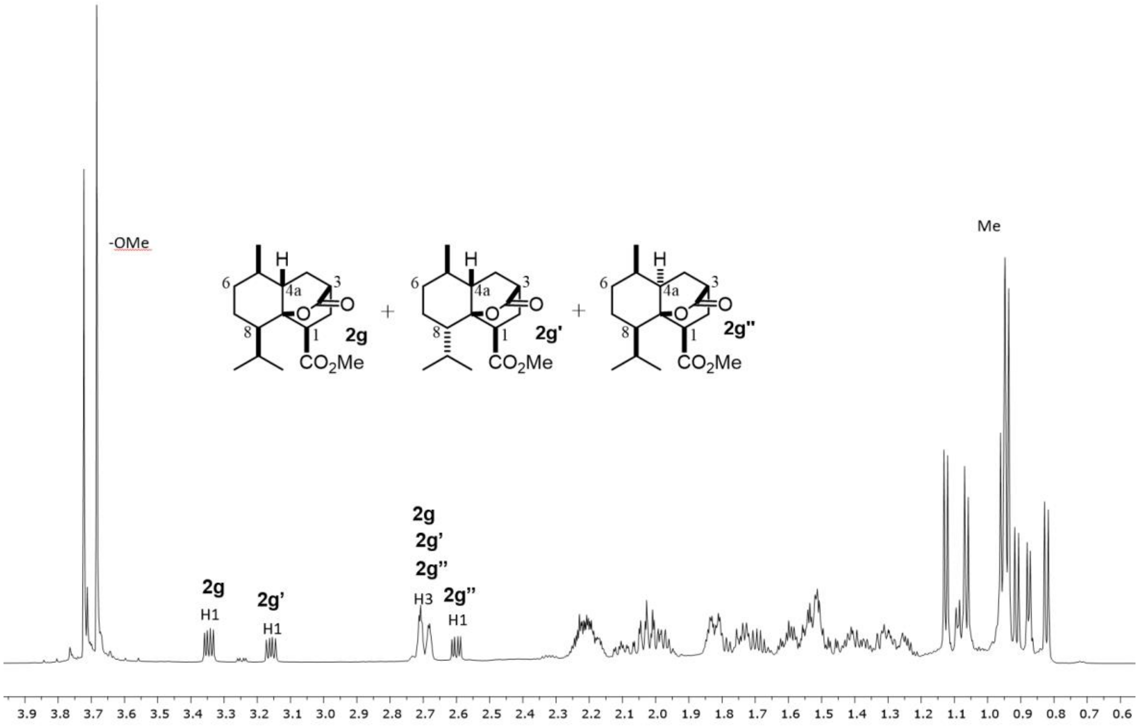

Figure S18. ^{1}H NMR spectrum of decalins **2g**, **2g'** and **2g''** (600 MHz, $CDCl_3$).

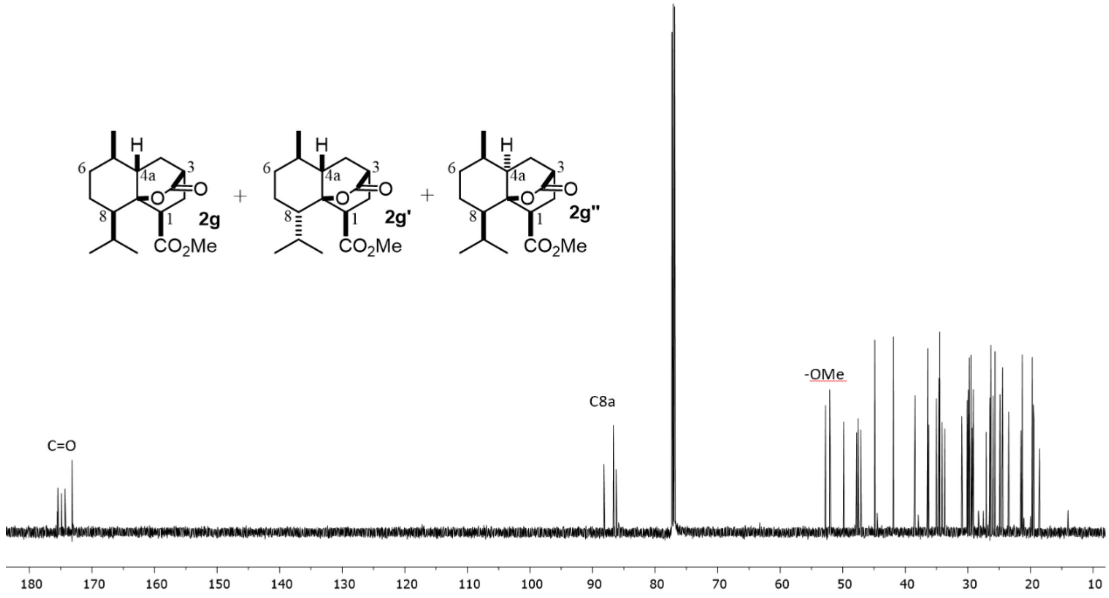

Figure S19. ^{13}C NMR spectrum of decalins **2g**, **2g'**, **2g''** (150 MHz, $CDCl_3$).

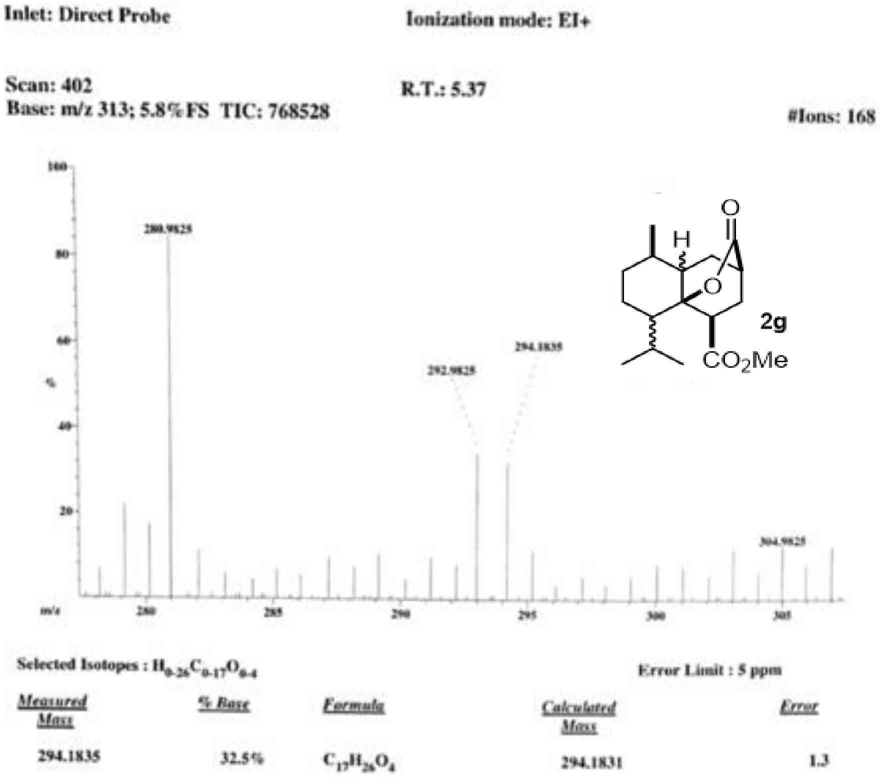

Figure S20. High-resolution mass spectrum of decalins **2g**, **2g'** and **2g''**.

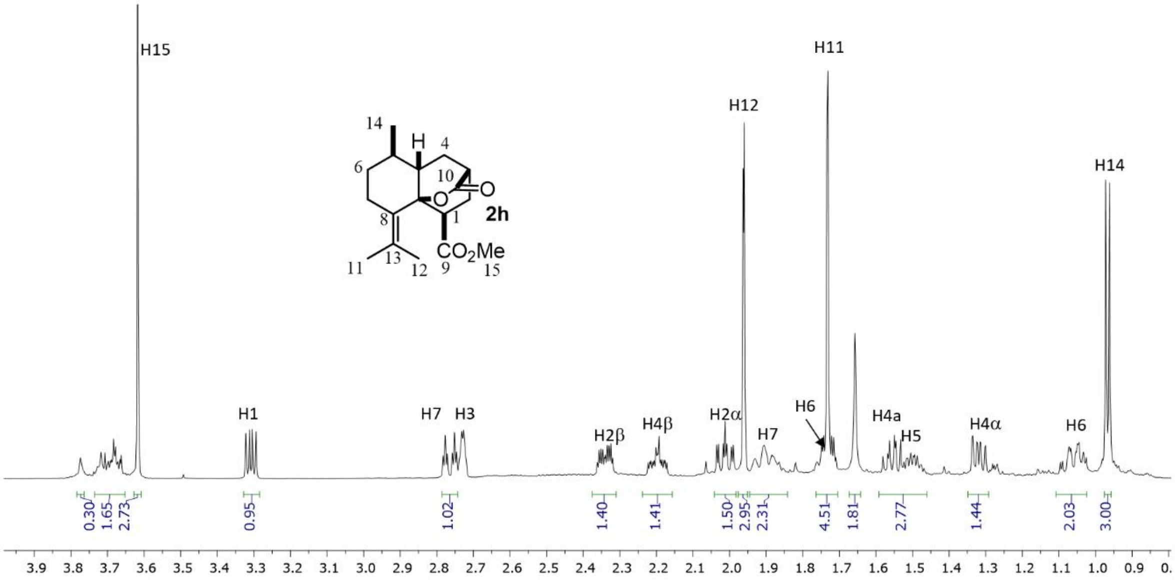

Figure S21. ^{1}H NMR spectrum of decalin **2h** (600 MHz, $CDCl_3$).

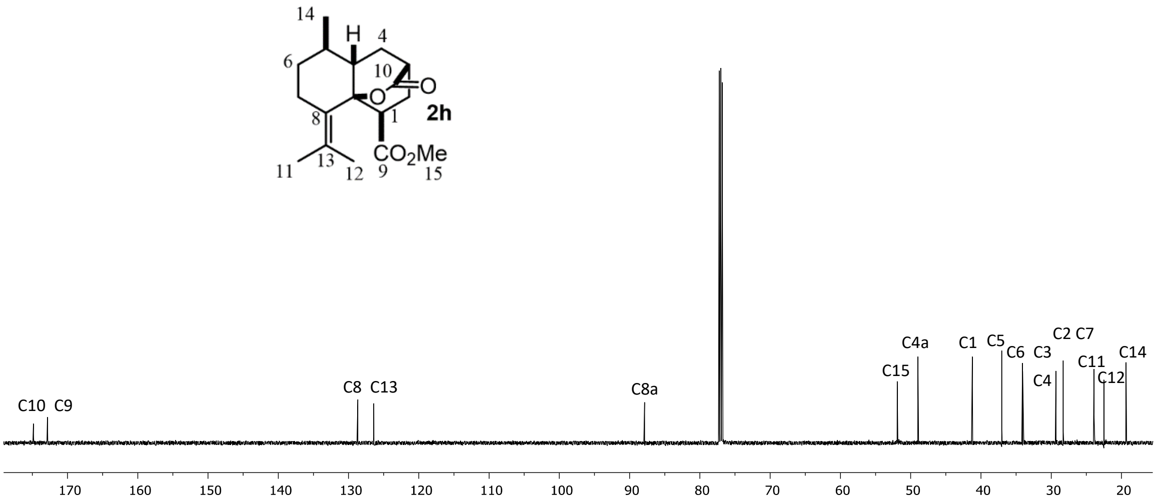

Figure S22. ^{13}C NMR spectrum of decalin **2h** (150 MHz, $CDCl_3$).

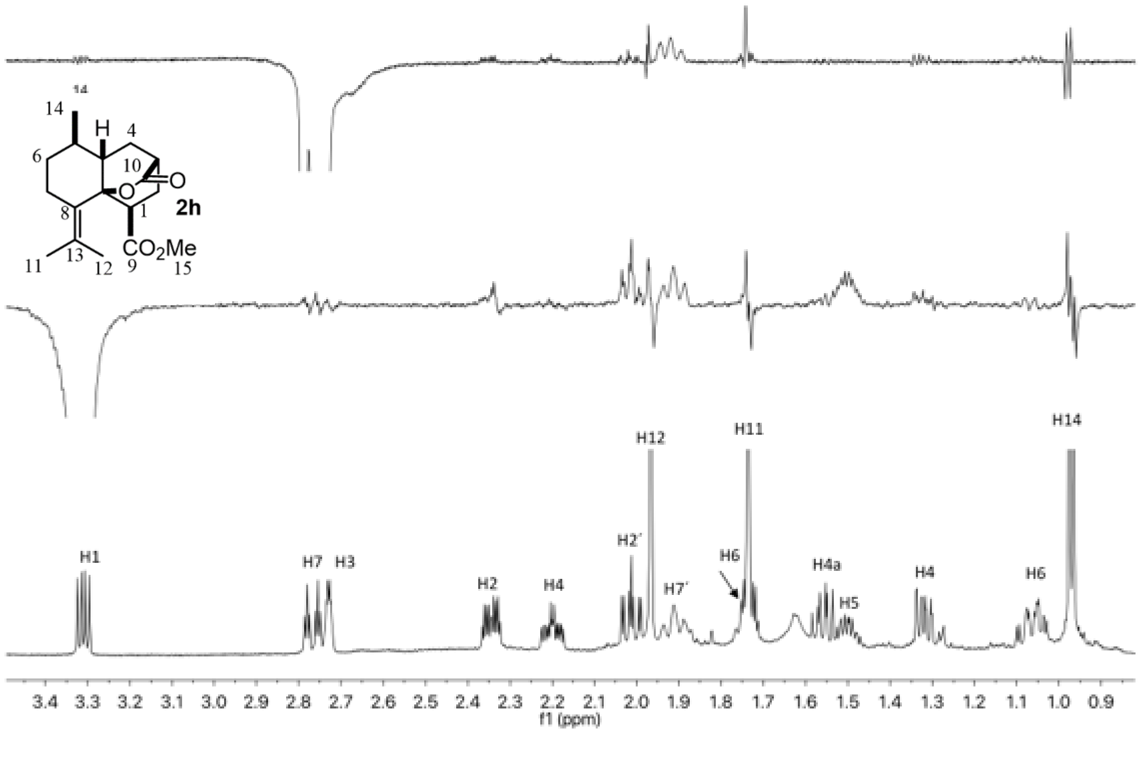

Figure S23. NOE spectrum of decalin **2h** (600 MHz, $CDCl_3$).

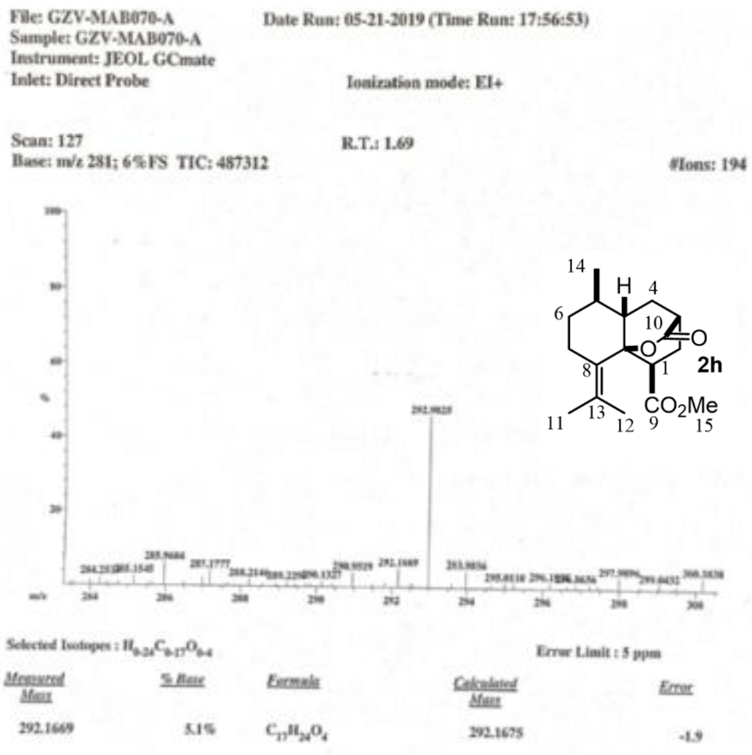

Figure S24. High-resolution mass spectrum of decalin **2h**.

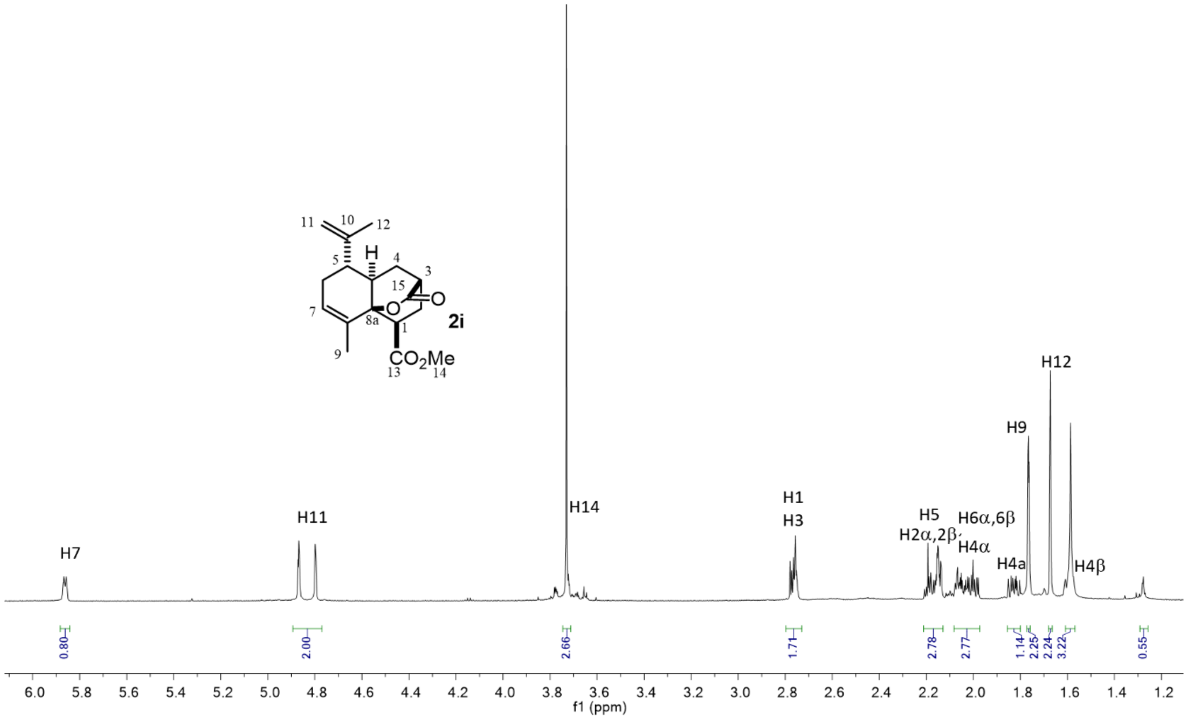

Figure S25. ^{1}H NMR spectrum of decalin **2i** (600 MHz, $CDCl_3$).

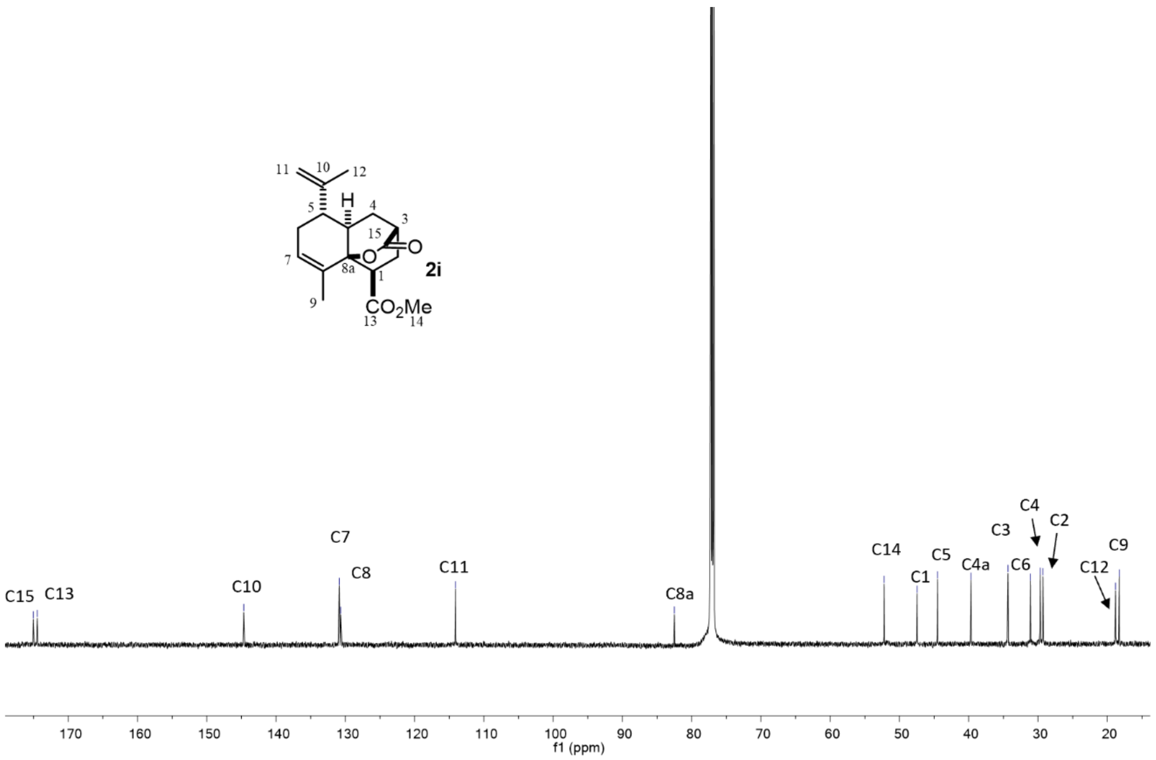

Figure S26. ^{13}C NMR spectrum of decalin **2i** (150 MHz, $CDCl_3$).

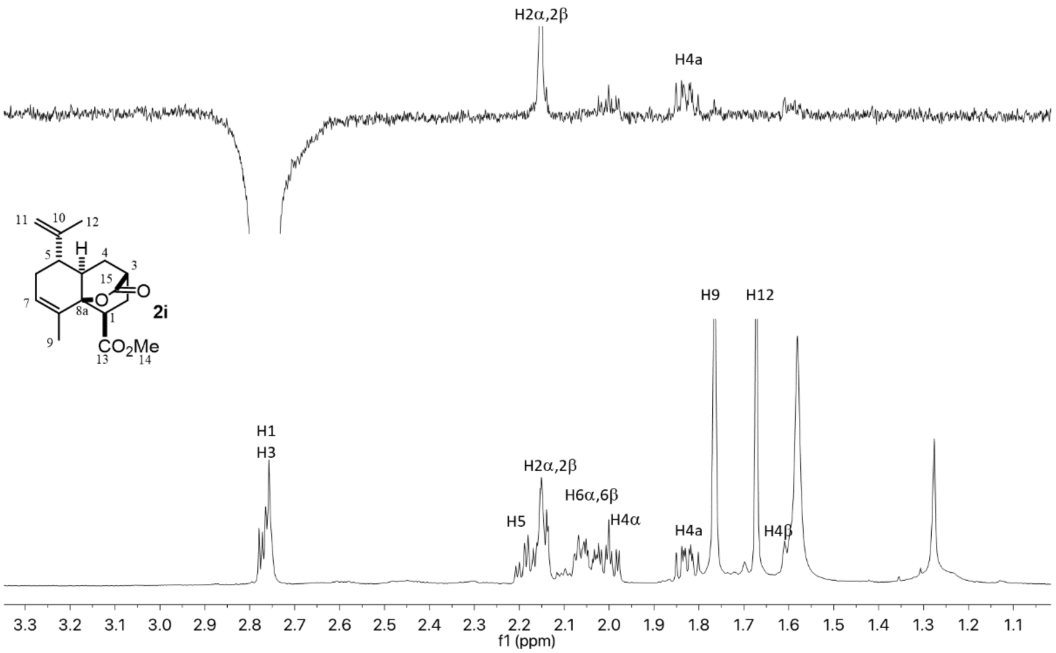

Figure S27. NOE spectrum of decalin **2i** (600 MHz, $CDCl_3$).

File: MAB087A-GZV
Sample: MAB087A-GZV
Instrument: JEOL GCmate
Inlet: Direct Probe

Date Run: 08-06-2019 (Time Run: 13:57:35)

Ionization mode: EI+

Scan: 287
Base: m/z 290; 10.2%FS TIC: 443600

R.T.: 3.79

#Ions: 169

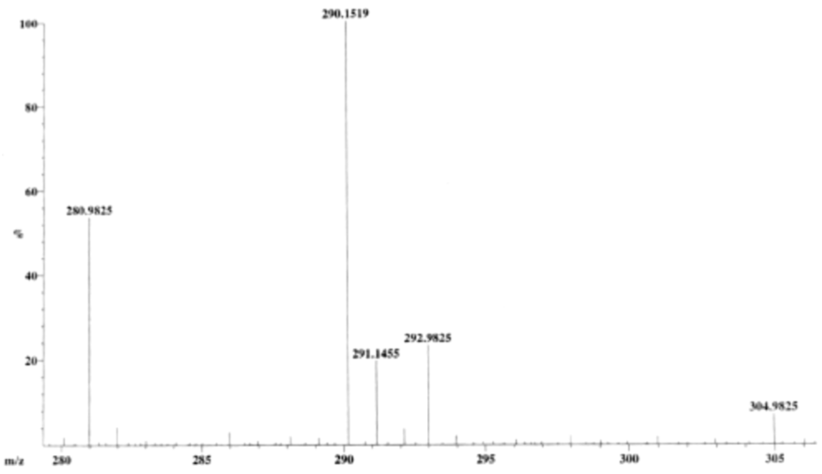

Selected Isotopes : $C_{0-17}O_{0-4}H_{0-22}$

Error Limit : 5 ppm

Measured Mass	% Base	Formula	Calculated Mass	Error
290.1519	100.0%	$C_{17}H_{22}O_4$	290.1518	0.3

Figure S28. High-resolution mass spectrum of decalin **2i**.

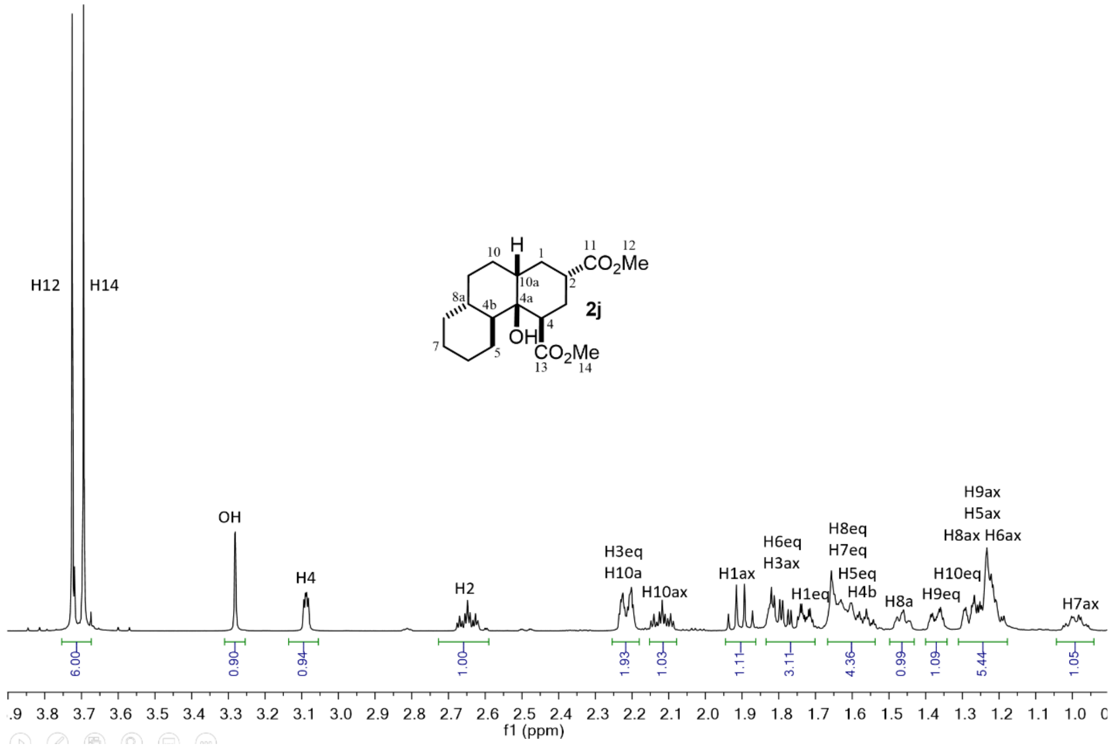

Figure S29. ^{1}H NMR spectrum of phenantrene derivative **2j** (600 MHz, $CDCl_3$).

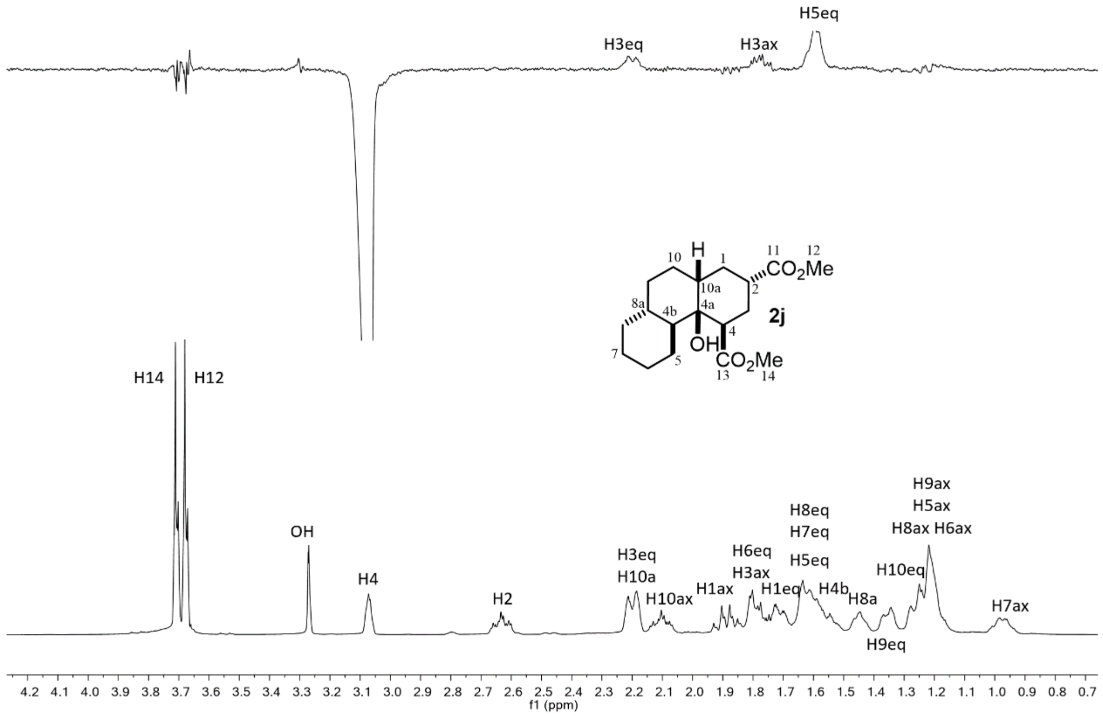

Figure S30. NOE spectrum of phenantrene derivative **2j** (600 MHz, CDCl3).

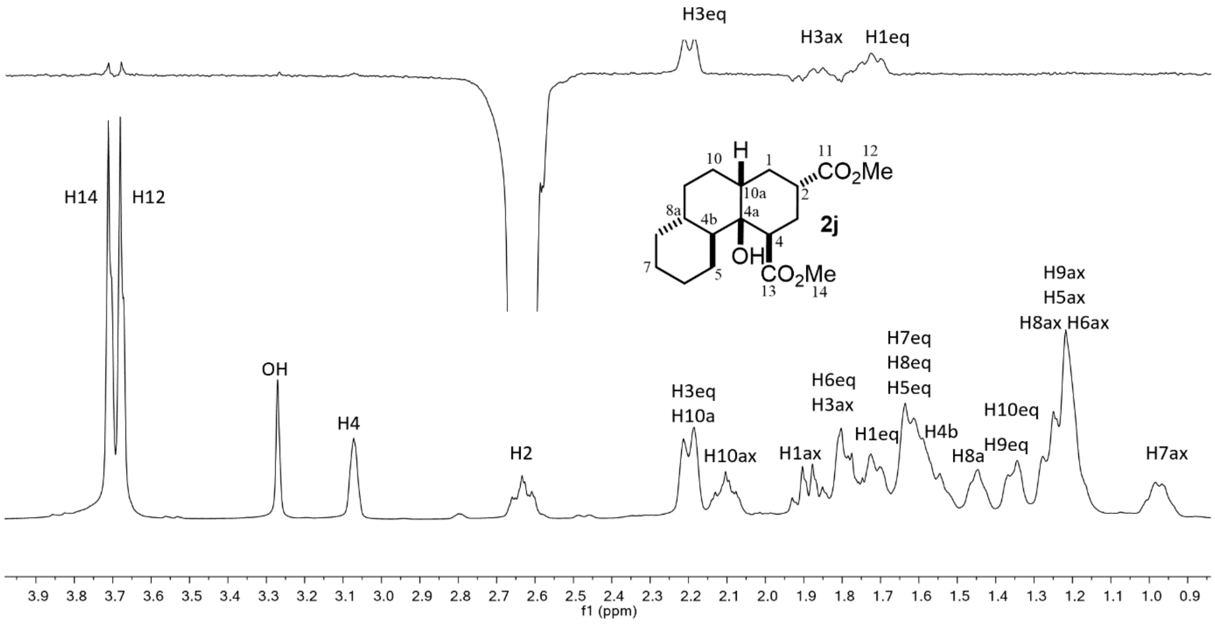

Figure S31. nOe spectrum of phenantrene derivative **2j** (600 MHz, CDCl3).

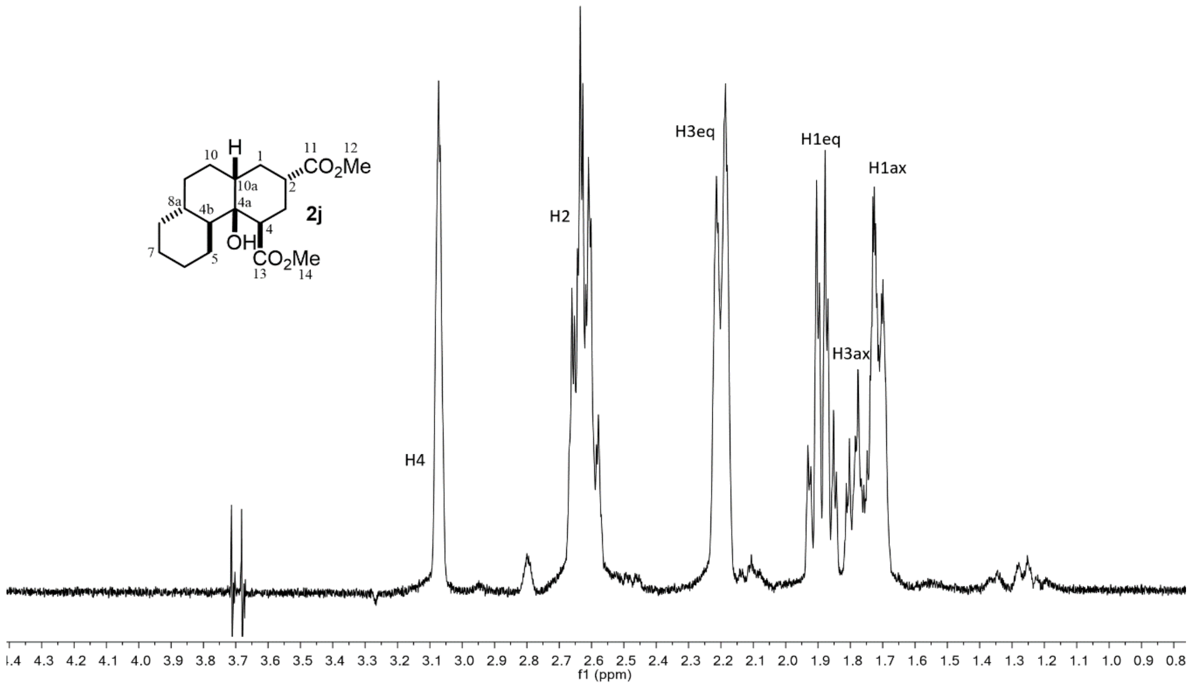

Figure S32. zTOCSY 1D spectrum of phenantrene derivative **2j** (600 MHz, CDCl3).

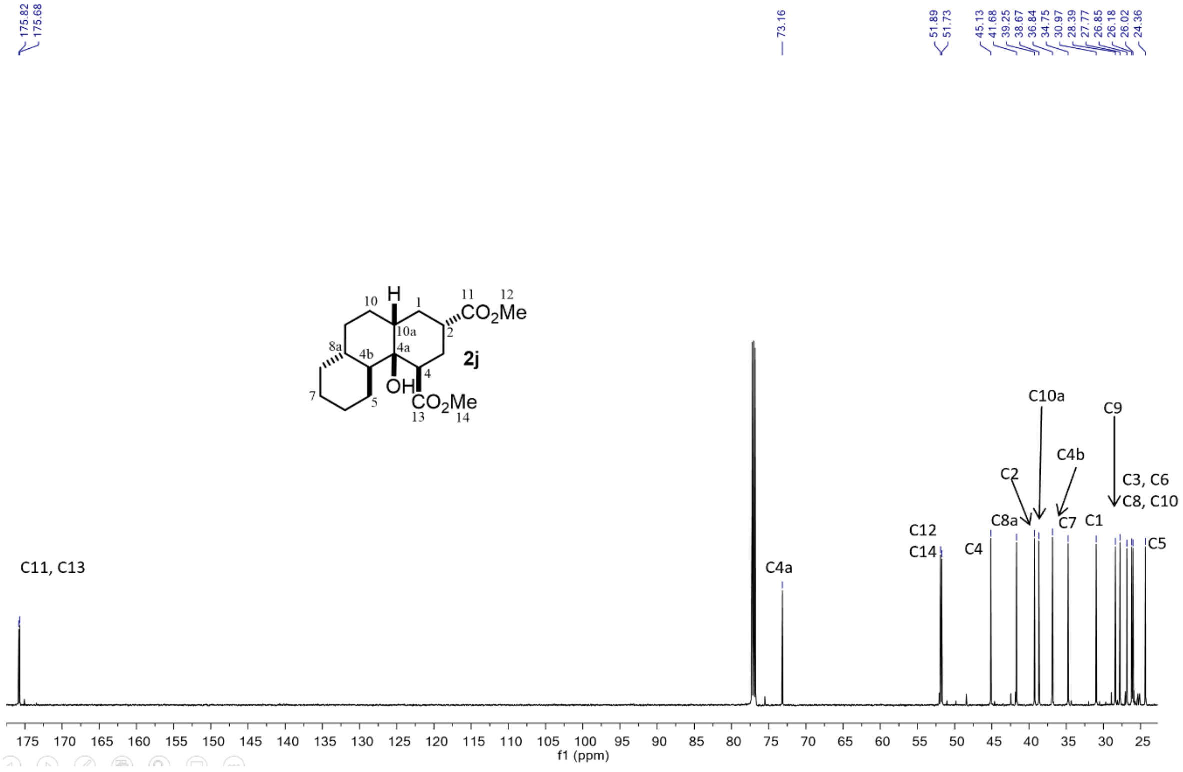

Figure S33. ^{13}C NMR spectrum of phenantrene derivative **2j** (150 MHz, $CDCl_3$).

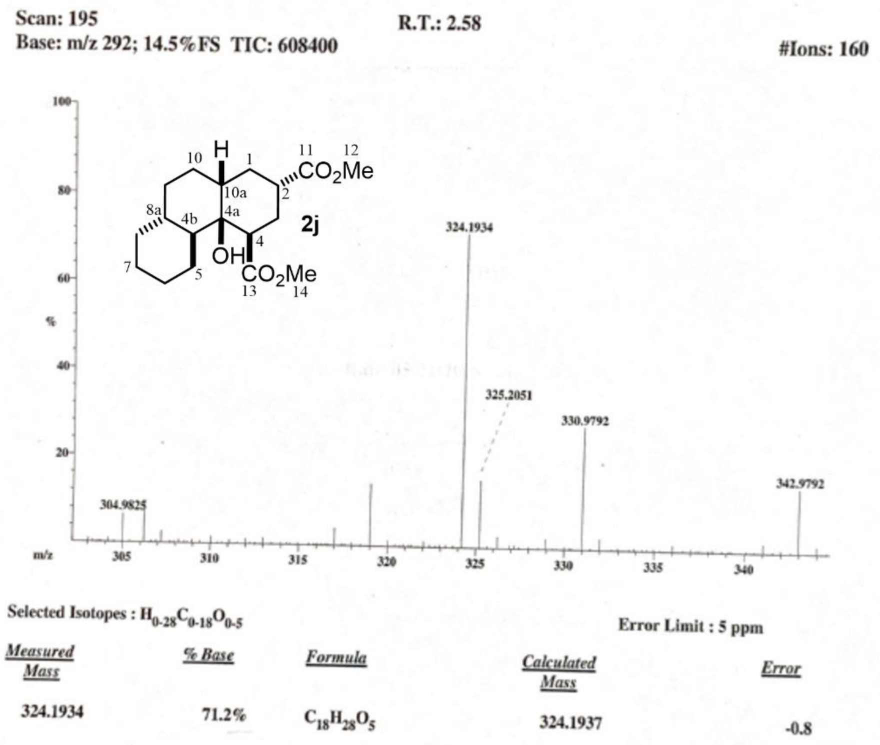

Figure S34. High-resolution mass spectrum of phenantrene derivative **2j**.

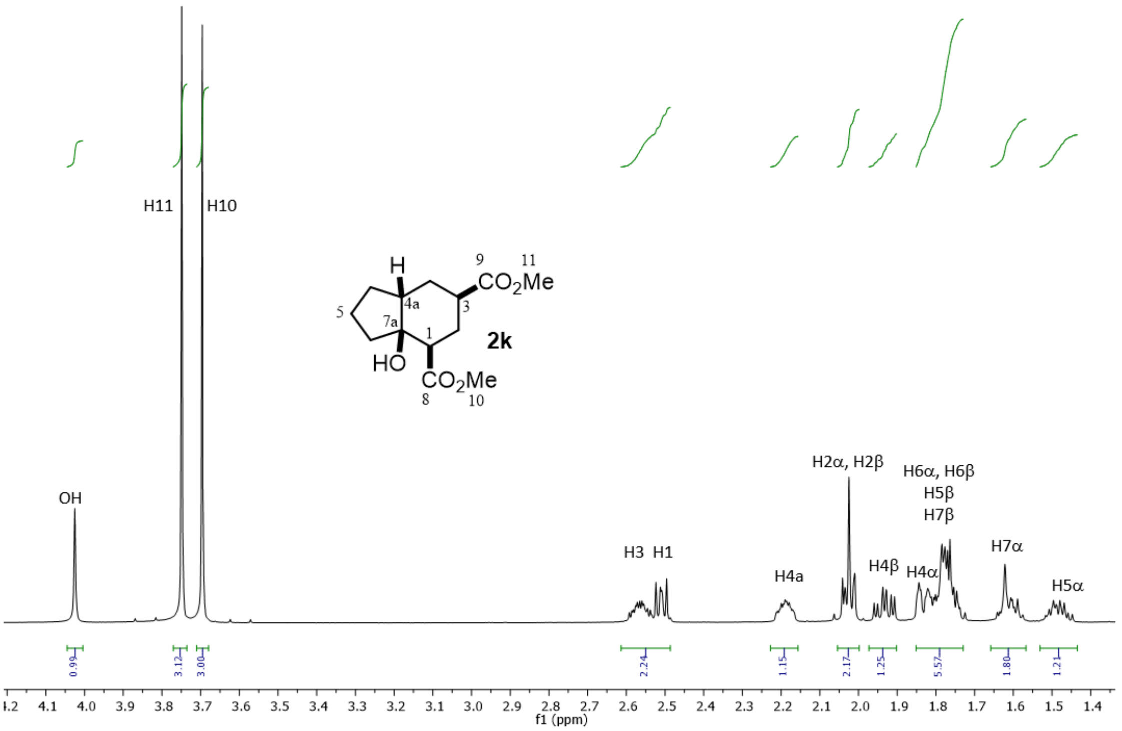

Figure S35. ^{1}H NMR spectrum of hydrindane **2k** (600 MHz, $CDCl_3$).

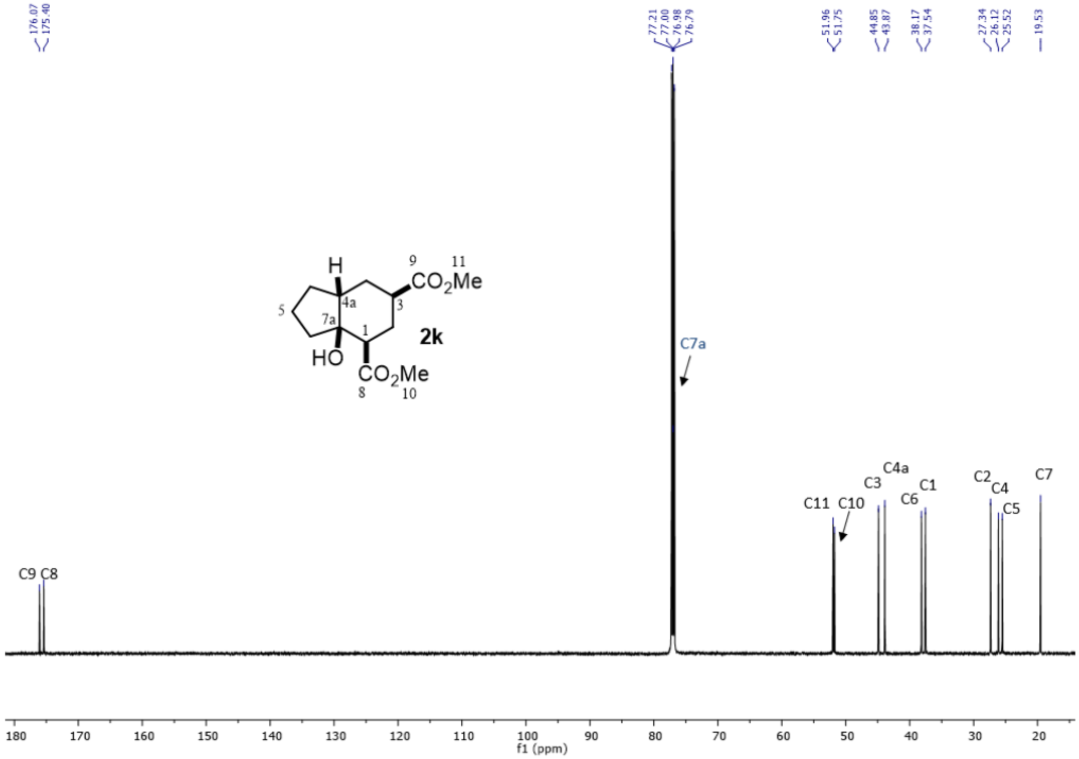

Figure 36. ^{13}C NMR spectrum of hydrindane **2k** (150 MHz, $CDCl_3$).

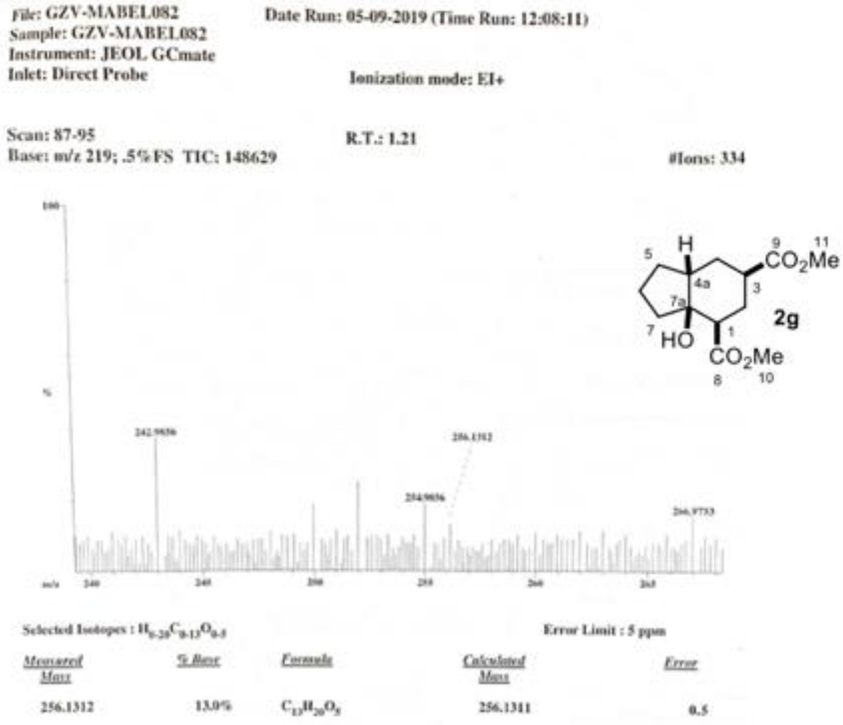

Figure S37. High-resolution mass spectrum of hydrindane **2k**.

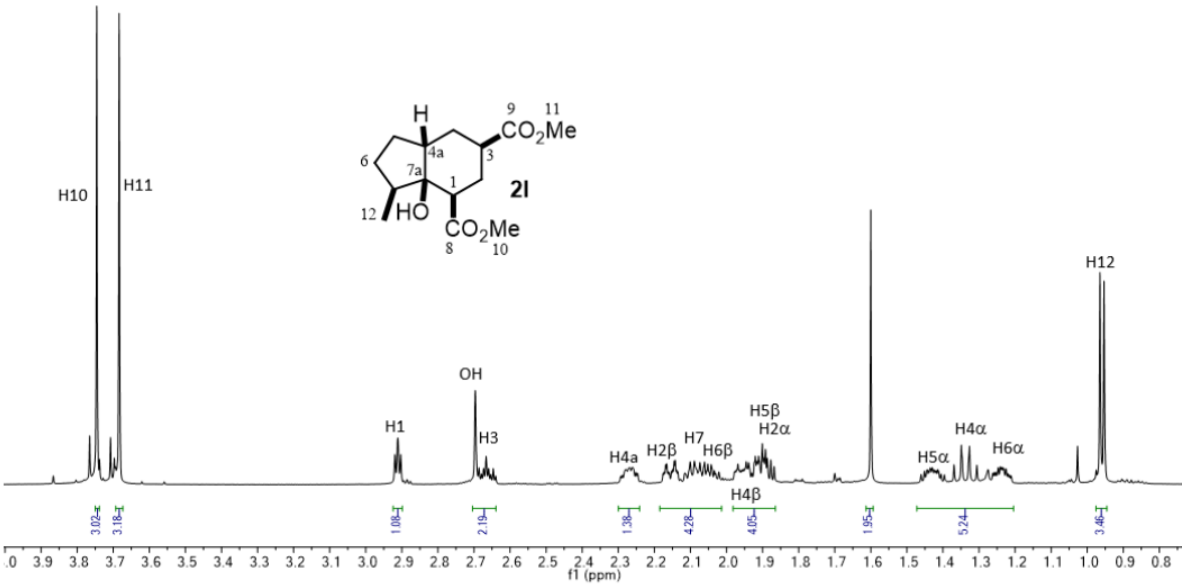

Figure S38. ^{1}H NMR spectrum of hydrindane **2l** (600 MHz, $CDCl_3$).

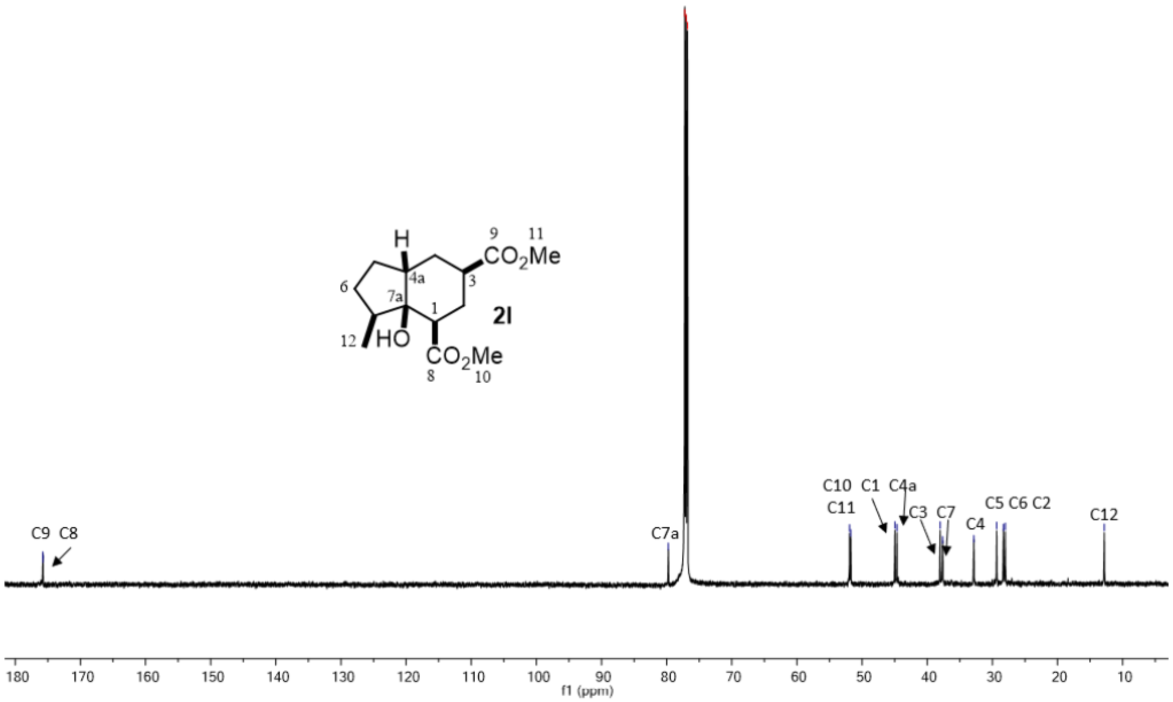

Figure S39. ^{13}C NMR spectrum of hydrindane **2l** (150 MHz, $CDCl_3$).

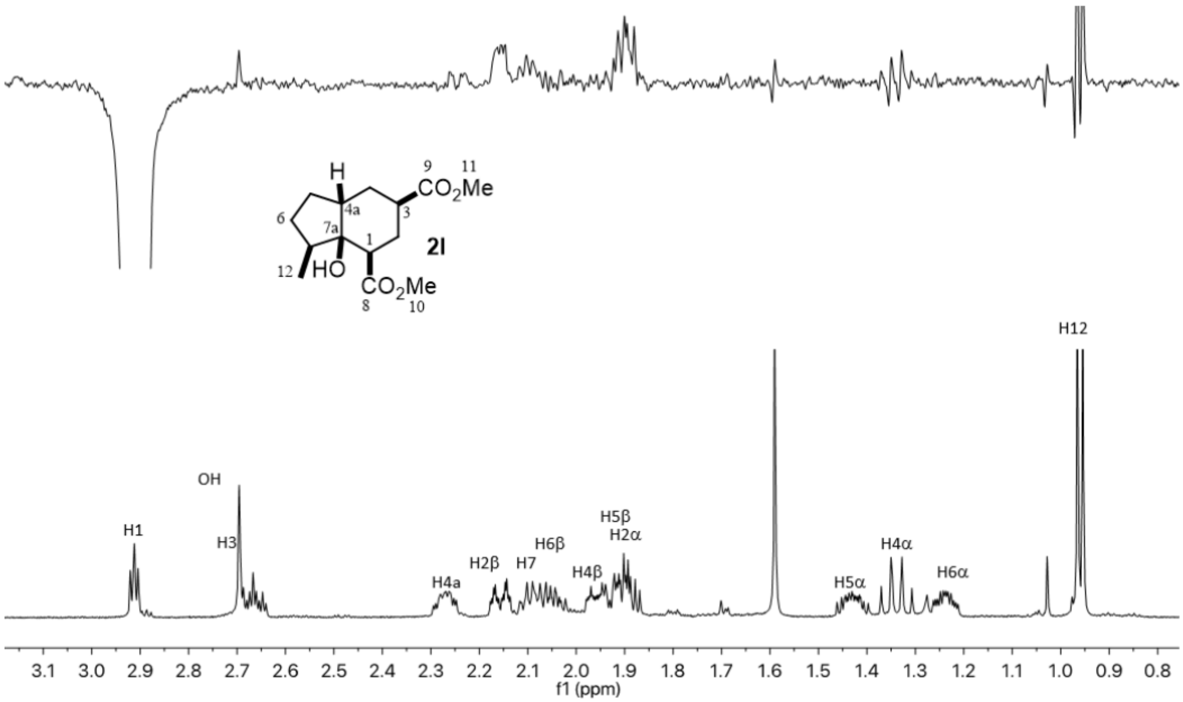

Figure S40. NOE spectrum of hydrindane **2l** (600 MHz, $CDCl_3$).

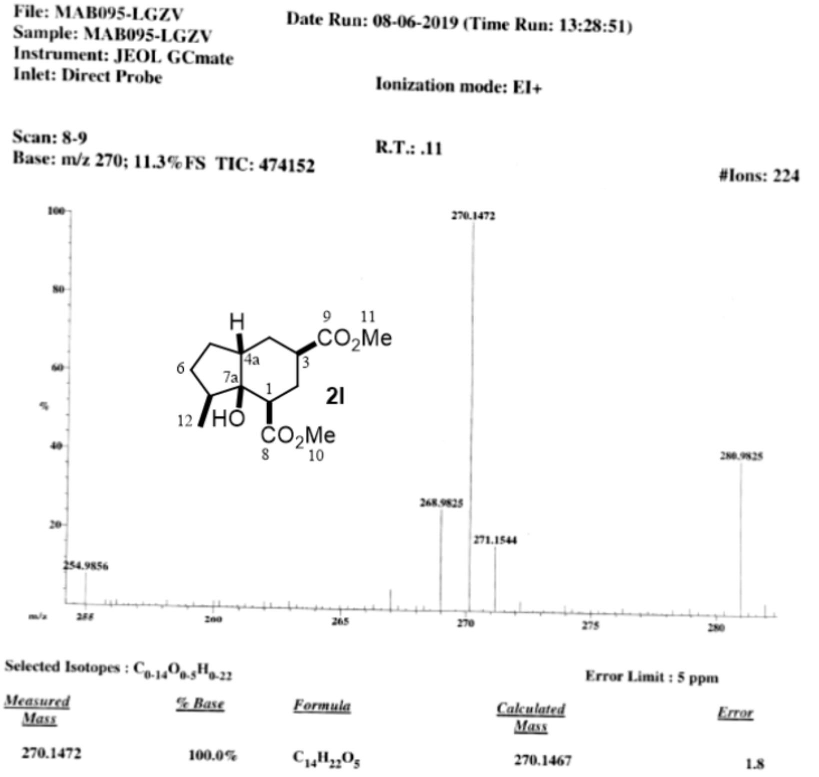

Figure S41. High-resolution mass spectrum of hydrindane **2l**.

3. Tables S4-S6. ^{1}H NMR parameters expected and calculated for decalins 2a, 2e, 2h, 2i, 2c and hydrindanes 2k and 2l.

Table S4. 1H NMR parameters expected and calculated for decalins 2a, 2e.

Decalin	Coupled protons	Dihedral angle (°)	***Jexpected* (Hz)**	***Jsimulated* (Hz)**	**δ(proton)**
**2a**	H1-H2ax	189	12.1	13.0	2.616 (H-1)
	H1-H2eq	304	3.8	3.8	
	H2eq-H3	290	1.8	2.3	2.103 (H-2eq)
	H2ax-H3	45	5.3	5.2	2.152 (H-2ax)
	H3-H4eq	70	1.8	2.0	2.787 (H-3)
	H3-H4ax	315	5.3	5.0	
	H4eq-H4a	59	3.3	3.0	1.709 (H-4eq)
	H4ax-H4a	175	12.3	12.2	1.842 (H-4ax)
	H4a-H5ax	185	12.3	12.9	1.8276 (H-4a)
	H4a-H5eq	302	3.5	3.0	2.1829 (H-5eq)
	H2eq-H2ax			-13.1	
	H4ax-H4eq			-13.1	
	H2eq-H4eq			-2.0	
**2e**	H1-H2eq	305	3.9	3.6	2.4281 (H-1)
	H1-H2ax	188	12.1	13.0	
	H2eq-H3	54	4.1	3.9	2.085 (H-2eq)
	H2ax-H3	171	12.1	12.5	2.185 (H-2ax)
	H3-H4eq	304	3.8	3.8	2.513 (H-3)
	H3-H4ax	188	12.2	12.5	
	H4eq-H4a	60	3.2	3.0	1.743 (H-4eq)
	H4ax-H4a	177	12.3	12.9	1.893 (H-4ax)
	H4a-H5eq	296	2.7	3.0	1.415 (H-4a)
	H4a-H5ax	179	12.4	12.9	
	H2ax-H2eq			-13.0	
	H2eq-H4eq			-0.9	
	H4ax-H4eq			-12.6	

Table S5. 1H NMR parameters expected and calculated for decalins 2i, 2h.

Lactone	Coupled protons	Dihedral angle (°)	*Jexpected* (Hz)	*Jsimulated* (Hz)	δ(proton)
**2i**	H1-H2α	4.4	10.3	10.0	2.769 (H-1)
	H1-H2β	246	3.2	2.5	
	H2α-H3	291	1.9	1.7	2.14 (H-2α)
	H2β-H3	48	4.8	4.5	2.1549 (H-2β)
	H3-H4α	55	3.7	4.0	2.7582 (H-3)
	H3-H4β	298	2.7	2.4	
	H4α-H4a	20	9.3	9.7	2.0009 (H-4α)
	H4β-H4a	138	7.6	7.4	1.593 (H-4β)
	H4a-H5α	171	11.5	12.0	1.8276 (H-4a)
					2.184 (H-5α)
	H2α-H2β			-14.0	
	H4α-H4β			-13.6	
	H2β-H4β			-2.5	
**2h**	H1-H2α	355	10.3	10.6	3.31 (H-1)
	H1-H2β	238	4.6	6.1	
	H2α-H3	299	3.1	2.8	2.013 (H-2α)
	H2β-H3	56	3.6	3.0	2.3435 (H-2β)
	H3-H4α	66	2.2	1.4	2.728 (H-3)
	H3-H4β	309	4.3	4.6	
	H4α-H4a	238	6.6	7.0	1.3204 (H-4α)
	H4β-H4a	345	9.8	10.4	2.20 (H-4β)
	H4a-H5α	175	12.1	11.0	1.558 (H-4a)
	H2α-H2β			-13.5	
	H4α-H4β			-13.09	
	H2β-H4β			-2.7	

Table S6. 1H NMR parameters expected and calculated for hydrindanes 2k and 2l.

Hydrindane	Coupled protons	Dihedral angle (°)	*Jexpected* (Hz)	*Jsimulated* (Hz)	δ(proton)
**2k**	H1-H2α	299	10.3	10.0	2.5096 (H-1)
	H1-H2β	181	4.7	4.0	
	H2α-H3	59.3	2.8	3.1	2.0219 (H-2α)
	H2β-H3	177.2	3.7	4.5	2.0258 (H-2β)
	H3-H4α	304	2.2	2.4	2.565 (H-3)
	H3-H4β	186	4.3	4.0	
	H4α-H4a	289	6.4	7.4	1.932 (H-4α)
	H4β-H4a	46.1	9.8	9.5	1.834 (H-4β)
	H4a-H5α	154.9	12.6	12.0	2.1885 (H-4a)
	H4a-H5β	37.1			2.1829 (H-5)
	H2α-H2β			-13.5	
	H4α-H4β			-13.2	
	H2β-H4α			-1.0	
**2l**	H1-H2α	52.3	10.3	10.6	2.9065 (H-1)
	H1-H2β	296	4.6	6.1	
	H2α-H3	183	3.1	2.8	2.013 (H-2α)
	H2β-H3	300	3.6	3.0	2.3435 (H-2β)
	H3-H4α	173.9	2.2	1.4	2.6613 (H-3)
	H3-H4β	57.9	4.3	4.6	
	H4α-H4a	192	6.6	7.0	2.0258 (H-4α)
	H4β-H4a	308	9.8	10.4	2.0219 (H-4β)
	H4a-H5α	90.4	12.1	11.0	2.265 (H-4a)
	H4a-H5β	335			
	H2α-H2β			-14.0	
	H4α-H4β			-13.6	
	H2β-H4β			-1.9	

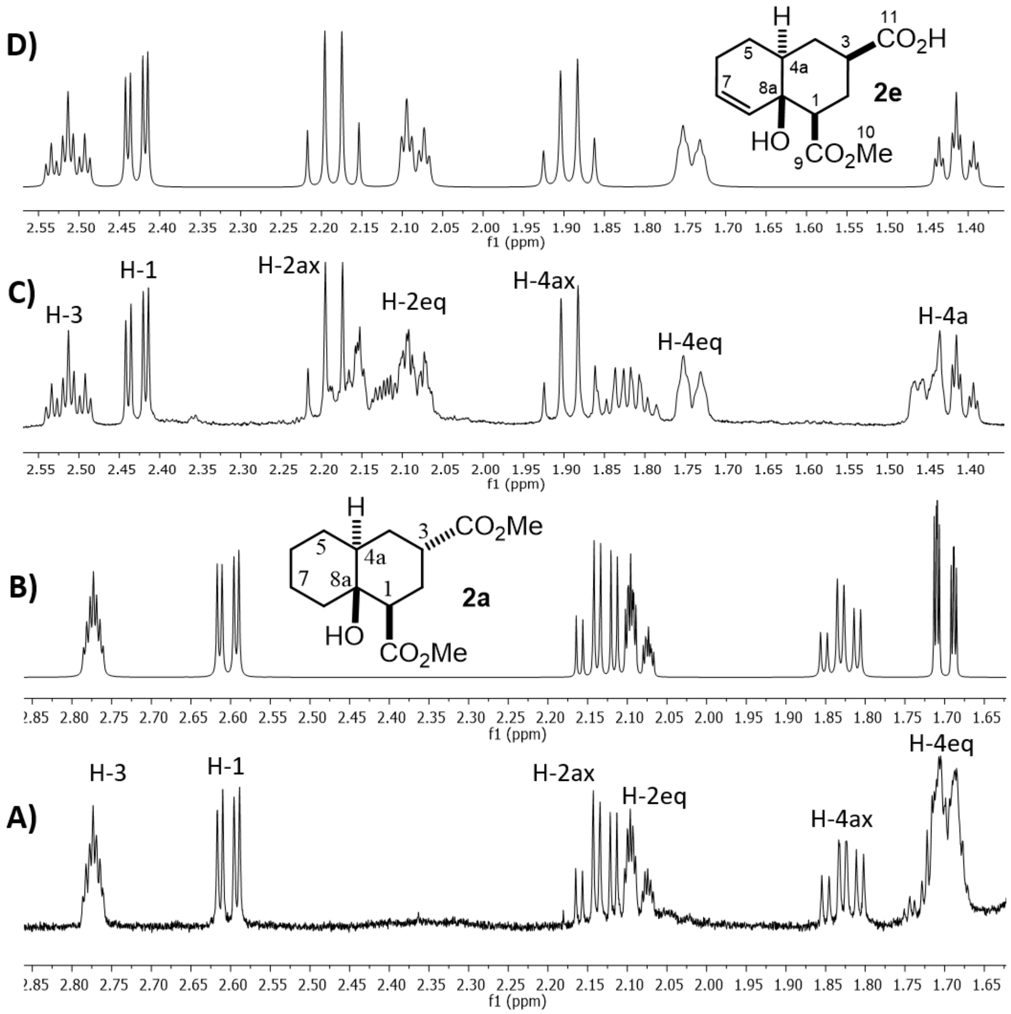

Figure S42. Experimental (A, C) and simulated (B, D) ^{1}H NMR spectra of decalins **2a** and **2e**, respectively. Full ^{1}H NMR simulated parameters were considered only for shown protons.

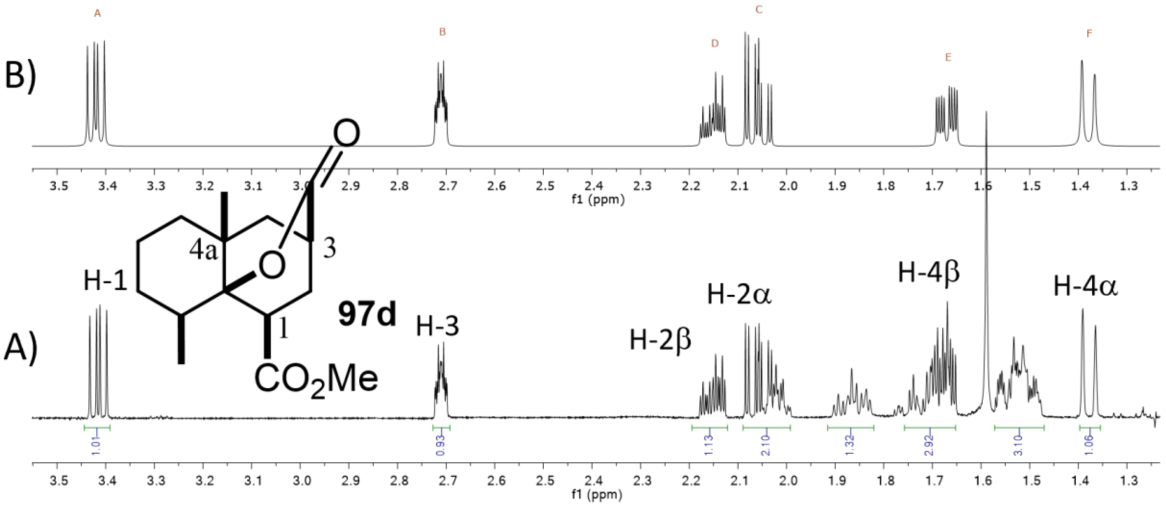

Figure S43. Experimental (A) and simulated (B) ^{1}H NMR spectra of decalin **2d**.
Full ^{1}H NMR simulated parameters were considered only for shown protons.

Table S7. 1H NMR parameters expected and calculated for decalins 2d.

Decalin	Coupled protons	Dihedral angle (°)	***Jexpected*** **(Hz)**	***Jsimulated*** **(Hz)**	***δ*(proton)**
**2d**	H1-H2α	345	9.8	10.55	3.42 (H-1)
	H1-H2β	224	7.0	6.88	
	H2α-H3	304	3.4	3.45	2.06 (H-2α)
	H2β-H3	62	2.7	2.34	2.15 (H-2β)
	H3-H4α	75	1.36	1.05	2.71063 (H-3)
	H3-H4β	318	5.85	5.34	1.67 (H-4β)
	H4α-H4β			-13.1	1.38 (H-4α)
	H2α-H2β			-13.1	
